# Valid Presumption of Shiga Toxin-Mediated Damage of Developing Erythrocytes in EHEC-Associated Hemolytic Uremic Syndrome

**DOI:** 10.3390/toxins12060373

**Published:** 2020-06-04

**Authors:** Johanna Detzner, Gottfried Pohlentz, Johannes Müthing

**Affiliations:** Institute of Hygiene, University of Münster, 48149 Münster, Germany; Johanna.Detzner@ukmuenster.de (J.D.); pohlentz@uni-muenster.de (G.P.)

**Keywords:** AB_5_ toxin, red blood cells, developing erythrocytes, EHEC, erythropoiesis, Gb3Cer, Gb4Cer, glycosphingolipids, hemolytic anemia

## Abstract

The global emergence of clinical diseases caused by enterohemorrhagic *Escherichia coli* (EHEC) is an issue of great concern. EHEC release Shiga toxins (Stxs) as their key virulence factors, and investigations on the cell-damaging mechanisms toward target cells are inevitable for the development of novel mitigation strategies. Stx-mediated hemolytic uremic syndrome (HUS), characterized by the triad of microangiopathic hemolytic anemia, thrombocytopenia, and acute renal injury, is the most severe outcome of an EHEC infection. Hemolytic anemia during HUS is defined as the loss of erythrocytes by mechanical disruption when passing through narrowed microvessels. The formation of thrombi in the microvasculature is considered an indirect effect of Stx-mediated injury mainly of the renal microvascular endothelial cells, resulting in obstructions of vessels. In this review, we summarize and discuss recent data providing evidence that HUS-associated hemolytic anemia may arise not only from intravascular rupture of erythrocytes, but also from the extravascular impairment of erythropoiesis, the development of red blood cells in the bone marrow, via direct Stx-mediated damage of maturing erythrocytes, leading to “non-hemolytic” anemia.

## 1. Introduction

The primary objective of the review is to improve our understanding of the clinical scenario of the hemolytic uremic syndrome (HUS) from a more mechanistic and biochemical point of view that focuses on the hemolytic anemia of patients suffering from infections of highly human pathogenic enterohemorrhagic *Escherichia coli* (EHEC). HUS-associated anemia is considered as the outcome of obstruction of vessels, which exert mechanical stress to circulating red blood cells when squeezing through narrowed microvessels, resulting in disruption and hence the loss of erythrocytes. However, the precise mechanisms that underly the hematologic impairments are largely unknown. We collate in this review previous and recent findings that suggest the erythropoietic system in the human bone marrow as an important target of Shiga toxins (Stxs), which are the major virulence factors of EHEC. Before going into the details of Stx-mediated injury of erythropoietic cells, we provide a few chapters in the beginning of the review looking beyond the horizon and shedding light on explanatory background knowledge related to the topic of the review. This might be helpful for understanding the main chapter dealing with the Stx-mediated damage of developing erythrocytes that are supposed to be connected to HUS-associated hemolytic anemia.

We start our review with the description of the mammalian hematopoietic system that represents the cell factory producing all the different types of mature blood cells being continuously generated in the bone marrow of skeletal bones. The general explanation of hematopoiesis leads to a detailed portrayal of erythropoiesis, including the various developmental stages of erythrocyte maturation controlled by erythropoietin (EPO). Next, we supply an updated overview of the current practice and improvements of the ex vivo production of developing erythrocytes, followed by a brief outline about some known prokaryotic pathogens and bacterial toxins that specifically harm human mature and/or developing red blood cells. Then, the review continues with a short historical reflection on the discovery of globo-series glycosphingolipids (GSLs) of human erythrocytes with an emphasis on the cardinal Stx receptors. This paragraph is supplemented by explanations of their chemical structure and highlights the differences between erythrocytes on the one hand and closely related myeloid and lymphoid cells on the other hand with regard to their distinct GSL profiles. The ensuing chapter deals at first with an evolutionary aspect of how Stx has developed as a primordial bacterial weapon against eukaryotic predators. Then, we describe the life-threatening diseases caused by EHEC and how Stx, the main virulence factor of EHEC, damages well known human target cells such as renal and cerebral microvascular endothelial cells. The subsequent chapter lays emphasis on the flexible shape and deformability of human erythrocytes, which can unscathedly pass through narrowed microvessels, and it provides a critical view on the common opinion of the mechanical rupture of red blood cells due to passage through constricted microvessels. Entering the main chapter of the review, we issue a synopsis of recent findings with respect to the direct Stx-mediated injury of developing erythrocytes. This includes clarification of the results by illustrations showing the morphological alterations occurring during the differentiation of hematopoietic stem/progenitor cells propagated in ex vivo cell cultures. Immunochemical detection depicts the concomitant changes in GSL expression as well as varied binding profiles of Stx2a, one of the clinically important Stx subtypes, toward globo-series GSLs further scrutinized by precise mass spectrometric analysis of their exact structures. The review ends with the conclusions that anemia can be at least in part the result of decreased red blood cell production due to Stx-mediated impairment of the erythropoiesis, which may lead to “non-hemolytic” anemia in HUS patients.

## 2. Hematopoiesis

**Figure 1:**
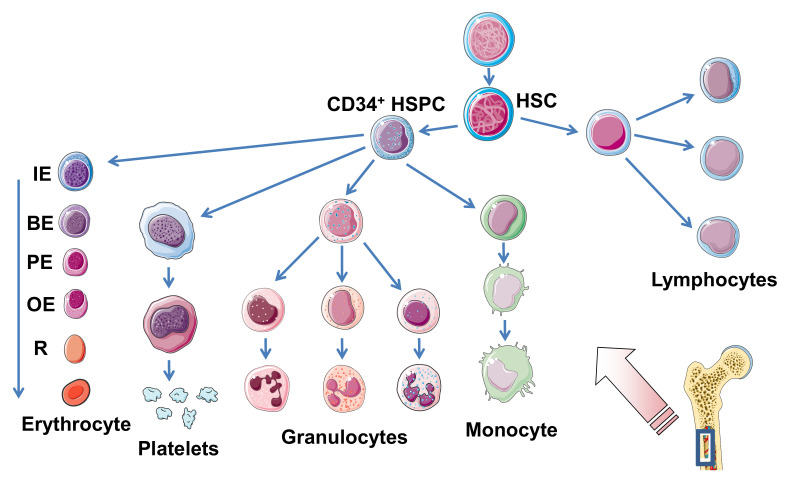
Scheme depicting the development of the diverse blood cells of the hematopoietic system that takes place in the bone marrow. Hematopoiesis starts from pluripotent hematopoietic stem cells (HSC). The erythroid and myeloid lineages originate from CD34^+^ hematopoietic stem/progenitor cells (HSPC). Differentiation (from left to right) of erythrocytes, megakaryocytes (platelet-forming cells), granulocytes (neutrophilic, eosinophilic, and basophilic, from left to right), monocytes (which further differentiate in tissues to adherent macrophages and/or dendritic cells), and small lymphocytes (T and B lymphocytes) as well as natural killer cells (large granular lymphocytes) representing the main groups of blood cells. IE, immature erythroblast (proerythroblast); BE, basophilic erythroblast; PE, polychromatophilic erythroblast; OE, orthochromatophilic erythroblast; R, reticulocyte. The figure was adapted from SERVIER MEDICAL ART (https://smart.servier.com) and modified in parts.

Mammalian hematopoiesis is a hierarchically organized process in which all types of mature blood cells are continuously generated from more primitive cells that lack any morphological evidence of differentiation [1], as shown in Figure 1. Enormous numbers of adult blood cells are constantly regenerated throughout life from hematopoietic stem cells (HSCs) through a series of progenitor cells aimed at keeping homeostasis of the cellular blood composition [2]. The hematopoiesis takes place in the bone marrow (medulla of the bone) as the primary site where multipotent HSCs reside in specialized microenvironments known as “niches” [3,4,5,6,7]. Hematopoiesis proceeds in long bones (femur and tibia) and other skeletal bone marrow-containing bones such as the ribs, the breastbone (sternum), the pelvic bone, and/or the vertebrae throughout life [8,9,10,11]. The simultaneous perpetuation of self-renewal and the generation of differentiated progeny is a characteristic feature of HSCs known as “asymmetric stem-cell division” [12]. Thus, HSC proliferation results in either self-renewal or differentiation into erythroid, myeloid (granulocyte–monocyte), and lymphoid precursor cells, thereby maintaining the balance between propagation and maturation as the linchpin of hematopoietic homeostasis [13]. Importantly, the proximate daughter cells cannot renew themselves and propagate along their committed pathway. Hematopoietic growth factors induce the mobilization and proliferation of HSCs and hematopoietic progenitor cells (HPCs), resulting in spatial and quantitative in vivo expansion of the hematopoietic tissue [14]. Certain hematopoietic growth factors that mobilize and regulate the proliferation and maturation of HSCs play key roles in hematopoiesis with potential for clinical use [15,16]. There are a number of colony-stimulating factors that are responsible for the specific mobilization of committed cells of the myeloid lineage, the stem cell factor (SCF), and various interleukins (IL) videlicet IL-2, IL-3, IL-5, and IL-7 [17,18]. Importantly, epigenetic modifications directly shape HSC developmental pathways, including the cellular maintenance of self-renewal and multilineage potential, lineage commitment, and aging [19,20]. Unraveling the molecular mechanisms that govern hematopoietic development in physiological and pathological conditions requires knowledge of the hematopoietic regulatory networks and their implication in gene expression to develop novel therapeutic concepts in regenerative medicine [21,22]. However, even key mechanisms such as DNA methylation, histone modifications, or non-coding RNAs inference underlying these modifications in the human genome are far from being fully understood [20]. The current knowledge of human hematopoietic development with respect to in vitro differentiation and available techniques as well as protocols that facilitate the generation of HSCs and their progeny has been recently reviewed [23]. In short, human pluripotent stem cells provide a vital opportunity to establish in vitro models of cell differentiation that will improve our understanding of the hematopoietic system. Novel approaches have been designed for generating progenitor populations intended for cell-based treatments and studying how specific hematopoietic cell subtypes undergo differentiation resulting in mature blood cells [23]. Concerning therapeutic interventions, EPO is applied for the treatment of anemia, and colony-stimulating factors are in use for the therapy of neutropenia, while other hematopoietic growth factors still need to demonstrate in vivo clinical relevance before reaching the market [24].

## 3. Erythropoiesis

The following chapters describe the various developmental stages of erythrocyte maturation being under the control of EPO, which regulates the proliferation and differentiation of erythrocyte progenitor cells, and the erythrocyte suicidal cell death termed “eryptosis”, which leads to deformed erythrocytes and may result in anemia.

### 3.1. Developmental Stages

Erythropoiesis of adult humans starts from hematopoietic stem/progenitor cells (HSPCs) residing mainly in the skeletal bone marrow where they develop to mature erythrocytes, traversing a series of consecutive erythroid progenitor cells. Erythropoiesis can be subdivided into three stages: early erythropoiesis, terminal erythroid differentiation, and reticulocyte maturation [25]. At early erythropoiesis, pluripotent HSCs proliferate and differentiate into committed erythroid progenitors videlicet erythroid burst-forming unit (BFU-E) and then erythroid colony-forming unit (CFU-E) cells. This is followed by initiation of the terminal erythroid differentiation of immature erythroblasts (proerythroblasts), which subsequently undergo sequential cell divisions to enter the stages of basophilic, polychromatophilic, and orthochromatophilic erythroblasts, which enucleate to become reticulocytes [25,26] (Figure 1). Erythroblasts progressively decrease in size, condense their nuclei, accumulate hemoglobin, and finally undergo enucleation to form reticulocytes until they become fully mature red blood cells (RBCs) [27]. More precisely, the immature erythroblast is the first cell that is morphologically recognizable in the erythroid lineage. Human immature erythroblasts (20–25 µm) possess large nuclei, which occupy 75%–80% of the cell volume (Figure 1). The smaller basophilic erythroblast (16–18 µm) is characterized by a nucleus being somewhat reduced in size, exhibiting coarser appearance and a more basophilic cytoplasm owing to the presence of ribosomes synthesizing hemoglobin. With the beginning of hemoglobin biosynthesis, the cytoplasm is dyeable with both basic and eosin stains being the reason for terming these progenitor cells polychromatophilic erythroblasts (“loving several colors”). They are smaller (12–15 µm), and the nucleus is more condensed compared to basophilic erythroblasts. Proceeding maturation results in orthochromatophilic erythroblasts, the last and smallest erythroid progenitors (10–15 µm) that possess chromatin-condensed nuclei [28,29] being incapable of cellular division. Nuclear expulsion in orthochromatophilic erythroblasts gives rise to reticulocytes (8–10 µm) that remain at first in the bone marrow, where they undergo further maturation for up to 48 h before becoming circulating fully developed erythrocytes [27,28]. Reticulocytes exhibit eponymous reticular (net-like) aggregates and retain organelles such as the mitochondria and polyribosomes. They leave the bone marrow via diapedesis through the bone marrow capillaries and enter the bloodstream, where they circulate for 24 to 48 h and constitute approximately 1% to 2% of the total erythrocyte count [27]. Erythrocytes have a normal lifespan of approximately 120 days in the blood stream and deliver oxygen from lungs to cells and tissues throughout the body by transportation bound to hemoglobin. Erythrocytes are small biconcave discs (6–8 µm) filled with hemoglobin containing no cellular organelles [27,30]. Importantly, in the progress of maturation, an erythroblast is converted from a cell with a large nucleus and a volume of about 900 fL to a flat enucleated disc with a volume of approximately 90 fL [31].

### 3.2. Erythropoietin

Erythropoietin (EPO) is the main humoral regulator of erythropoiesis that stimulates the proliferation and differentiation of erythroid precursor cells [32]. EPO is mainly produced by specialized pericytes in the kidneys [33] that wrap around the endothelial cells in the microcirculation. Its plasma concentration is essentially under control of the oxygen partial pressure in the circulation, regulating the production of RBCs [34]. A decrease in the partial pressure of O_2_ increases the activity of the hypoxia-inducible transcription factor, which in turn triggers EPO gene transcription [32]. Disorders of kidney function can lead to inadequate EPO production, and compromised release of EPO from the defective kidney with subsequent impairment of erythropoiesis is the primary cause of anemia in chronic kidney disease [35]. In this disease, pericytes transdifferentiate to myofibroblasts, and the EPO production subsequently decreases, leading to renal anemia [33]. Consequently, the treatment of renal anemia is still restricted to EPO-stimulating agents. EPO represents arguably the most successful drug spawned by the revolution in recombinant DNA technology [36]. However, the various available EPOs, notably the three generations of EPOs, can be misused by athletes. Significant advances have occurred in detecting EPO misuse and, currently, the World Anti-Doping Agency’s athlete biological passport with its hematological component has become an important but not infallible mechanism to identify heating athletes [37,38].

### 3.3. Eryptosis

Similar to the apoptosis of nucleated cells, erythrocytes may undergo eryptosis, a suicidal erythrocyte death characterized by cell shrinkage, cell membrane blebbing, and breakdown of the phospholipid asymmetry [39,40,41,42]. The disturbed membrane assembly results in phosphatidylserine exposure at the cell surface, which, in turn, mediates phagocytic recognition and the rapid clearance of deformed erythrocytes from the circulation in the liver [43]. Eryptosis is enhanced in a variety of clinical conditions including, among many others, HUS [44,45,46]. If compensation of eryptosis by enhanced erythropoiesis is not sufficient, clinically relevant anemia develops [42,47]. Beyond this, enhanced eryptosis shortens the lifespan of circulating erythrocytes and confers a procoagulant phenotype. This phenomenon has been tangibly implicated in the pathogenesis of anemia, impaired microcirculation due to the adhesion of eryptotic erythrocytes to the endothelial cells of the microvasculature, and prothrombotic risk associated with a multitude of clinical conditions [43].

## 4. Ex Vivo Generation of Developing Erythrocytes

Although blood transfusion is a vital therapy in carrying out and improving many medical and surgical applications, the ex vivo generation of RBCs for clinical transplantation appears on the horizon. Basic research on the dynamics of cellular differentiation markers and the employment of certain erythropoietic growth factors and cytokines paved the way for future biotechnological production in bioreactors on industrial scale. These items are briefly outlined in the following subparagraphs.

### 4.1. Blood Transfusion

Blood transfusion is an indispensable part of modern medicine in supporting numerous clinical therapies [48,49,50]. However, the complete procedure from blood collection to administration faces a number of concerns and challenges to overcome that need to be addressed [51,52]. Major handicaps are the paucity of appropriate donors, possible transfusion-transmitted infections, new emerging pathogens or pathogen-derived toxic compounds, and the overall costs of the transfusion procedure eliciting an increasing demand for artificial blood [53]. Thus, the ex vivo production of transfusable RBCs from HSCs provides a solution for deficiencies in blood transfusion and has met scientific, medical, and industrial interest [54,55]. Significant progress in exploring erythropoiesis paved the way toward the realization of this task and improvements in refining the ex vivo cell production of erythropoietic cells will overcome obstacles of the currently available methods in the near future [52].

### 4.2. Ex Vivo Generation of Cells of the Erythroid Lineage

Ex vivo expansion of HSCs for clinical use has been recognized as a very promising approach for hematotherapy, since HSCs are known to reconstitute the hematopoietic system in disease-related bone marrow failure and bone marrow aplasia [56]. Bone marrow aspirates, mobilized peripheral blood, and umbilical cord blood have developed as graft sources for HSPCs for stem cell transplanation and other cellular therapeutics [57,58,59]. Besides embryonic stem cells and induced pluripotent stem cells, primary HSCs have shown the potential to produce RBCs, giving rise to possible clinical applications [55,60,61]. Advances in unraveling the molecular and cellular mechanisms as well as the metabolic pathways governing erythrocyte development and regeneration [26,62,63] have encouraged the optimization of the ex vivo generation of erythroid cultures. HSCs can be mobilized from the bone marrow into the peripheral blood using hematopoietic colony-stimulating factors, allowing the convenient harvest of these cells for clinical transplantation [64]. The ex vivo proliferation and differentiation of developing erythrocytes demands on external signals, such as EPO, SCF, and IL-3, allowing for the regular production of mature and transfusable units of RBCs [65,66]. It is now possible to enrich for erythroid progenitors and precursors to a much greater extent than has been possible before with impact on application to regenerative medicine [67,68]. Stem cells can be accumulated, e.g., by the use of anti-CD34^+^ monoclonal antibodies [69]. CD34 is a transmembrane glycoprotein, which was first identified on HSPCs [70]. Moreover, CD34 is commonly applied as a target for the selection and enrichment of HSCs for bone marrow transplants, since CD34^+^ cells have been known for quite some time as being capable of reconstituting all hematopoietic lineages [71]. A number of systems have been developed to facilitate the isolation of these hematopoietic cell populations, including immunoaffinity columns, immunomagnetic beads, and submicroscopic beads. They are based on the utilization of commercially available monoclonal anti-CD34 antibodies and are suitable for the isolation of highly purified CD34^+^ cells from various hematopoietic sources [72,73,74,75]. Immunomagnetic beads conjugated with monoclonal anti-CD34 antibodies have been reported, allowing for the efficient isolation of CD34^+^ progenitor cells from peripheral blood with a degree of purity >90% by use of a magnetic cell selector [76]. However, CD34 is expressed at low frequency not only in cells from peripheral and umbilical cord blood, but also in cells derived from tissues of non-hematopoietic origin and is therefore considered as general marker for diverse progenitor cells [70,77].

### 4.3. Dynamics of Erythropoietic Markers Glycophorin A, CD36, and CD45

The normal development of RBCs is accompanied by the expression of a number of functionally distinct and stage-specific cell surface membrane proteins [78,79]. By examining their dynamic changes during ex vivo differentiation, the surface markers of maturing erythroid cells such as glycophorin A (GPA, CD235a) and the thrombin receptor CD36 [80] are associated with certain developmental stages, whereas the CD45 (common leukocyte antigen), a marker of the myeloid lineage, gets lost during proceeding erythroid differentiation. More specifically, GPA is a highly glycosylated transmembrane protein carrying mostly O-linked glycans [81,82] and exhibits an apparent molecular weight of approximately 39 kDa [83,84]. GPA is a renowned marker for the preceding maturation of erythroid cells during development from immature erythroblasts until the final stages of erythroid differentiation achieved in ex vivo cell cultures [25,85,86,87]. The CD36 transmembrane protein provides another useful marker to outline maturation [87]. CD36, also known as glycoprotein IV, is a highly glycoslyated integral membrane protein with an apprent molecular weight of 88 kDa [88]. Early erythroblast differentiation is accompanied by a rapid and progressive increase of CD36. Its expression is retained at intermediate levels and slightly decreases in the nucleated erythroid population along with a reduction in cell size [25,85,87]. On the other hand, the CD45 membrane protein is strongly expressed among all hematopoietic cells except for mature erythrocytes, which completely lack this protein [89]. CD45 is a leukocyte transmembrane glycoprotein with a molecular mass of about 200 kDa [90], harboring an intrinsic receptor-linked protein tyrosine phosphatase activity and playing a crucial role in the regulation of signal transduction in immune cells [91,92,93]. CD45 shows moderate to faint expression of early stage erythroblasts and disappears as cells develop from erythroid progenitors to more mature nucleated erythroid cells [66,87]. Importantly, innovative mass spectrometry-based proteomic analysis allows for the total molecular characterization of dynamic proteome changes that occurr during erythropoiesis. A comprehensive quantitative expression analysis of 6130 proteins has been performed, highlighting a breakpoint in the erythroid differentiation process at the basophilic stage of RBC development [94]. Proteomic analysis provides the foundation for future studies of disordered erythropoiesis that may correspond to the specific developmental stages of erythropoietic differentiation. Moreover, the mass spectrometric technology is capable of generating a wealth of data beyond the proteome. Of note, the novel mass spectrometry imaging of cells and tissue throughout erythroid differentiation ex vivo should be capable of precisely characterizing metabolic and lipidomic changes, opening new avenues for erythropoiesis research [95,96,97].

### 4.4. Biotechnological Aspects

The ex vivo generation of human RBCs from HSCs has been established, permitting the massive expansion of CD34^+^ stem cells by mimicking the marrow microenvironment. This has been done through the application of cytokines and the coculture of HSCs on stroma cells, reaching 100% conversion into mature RBCs coupled with the substantial amplification of CD34^+^ stem cells up to 1.95 × 10^6^-fold [98,99]. The described protocol comprises cell proliferation and erythroid differentiation under serum-free conditions in the presence of growth factors and emphasizes the impact of the ex vivo medullar microenvironment on the terminal maturation of erythroid cells, which can be adopted to HSCs from diverse sources: bone marrow, cord blood, or peripheral blood. Thus, the production of bio-engineered RBCs from stem cells ex vivo on the industrial level has become a possible alternative to classical transfusion products [86,100,101]. However, the major challenge requires biotechnological breakthroughs with respect to the efficacy and safety as well as the switch from two-dimensional production to large-scale three-dimensional bioreactors, allowing a cost-effective process to match the current prices of high-quality blood products. In addition, biological validation of cellular alterations resulting from a poorly controlled production process is needed as well as monitoring the quality of the transfusion products arising from new biotechnologies, assuming that the culture conditions may influence the quality of the cell products generated [100]. Nevertheless, the proof of principle for the transfusion of RBCs generated ex vivo under good manufacturing practice conditions has testified globally their quality and functionality [102]. An alternative resource for ex vivo produced erythrocytes as a continuous supply of RBCs are immortalized erythroid progenitor cell lines that are inducible to differentiate in vitro and are able to produce mature enucleated and transfusable RBCs [103,104,105,106].

## 5. Mature and Developing Erythrocytes as Targets for Pathogens and Bacterial Toxins

A few examples of pathogens, which are known to target mature or developing RBCs, are described in the next section, followed by mentioning some bacterial toxins with the potential to damage cells of the human erythroid lineage.

### 5.1. Pathogens That Target Human Mature or Developing Red Blood Cells 

RBCs can be harmed by infectious microorganisms and pathogen-released toxic compounds, resulting in hemolysis and associated hemolytic anemia. The invasion of RBCs by *Plasmodium falciparum*, the best known and most serious form of malaria, involves several erythrocyte-binding ligands of the heavily glycosylated glycophorins A, B, C, and D [107,108]. High rates of parasitemia in which >10% of RBCs are parasitized may cause significant hemolysis and anemia. The human–pathogenic parvovirus B19 is also a causative agent of anemia, showing a remarkable tropism for human erythroid progenitor cells, namely the erythroid burst-forming and colony-forming unit (BFU-E and CFU-E, respectively) cells that result in the viral suppression of erythropoiesis [109,110,111,112]. The B19 parvovirus targets the erythroid progenitors in the bone marrow by binding to the GSL globotetraosylceramide (Gb4Cer, globoside) [113]. Parvovirus B19 is highly tropic to human bone marrow, replicates only in erythroid progenitor cells, and may cause chronic anemia in case of persistent infection [114,115]. Large receptor-mediated structural changes of capsid rearrangements required for subsequent virus uptake [116] trigger cell death either by lysis or apoptosis, so anemia may develop [107,117]. Individuals negative for Gb4Cer are naturally resistant to infection with parvovirus B19 [118]. Interestingly, parvovirus was found to also bind to human myeloblasts at early myeloid differentiation that do express both globo- and neolacto-series GSLs, of which Gb4Cer represents the potential virus receptor [119].

### 5.2. Bacterial Toxins That Target Human Mature or Developing Red Blood Cells 

A number of bacteria-released toxins harms RBCs, causing intravascular hemolysis or the accelerated destruction of damaged RBCs via the liver and spleen [107]. The α-toxin of *Clostridium perfringens* induces the hemolysis of erythrocytes from various species due to its lipolytic enzymatic activities, phospholipase C, and sphingomyelinase, which preferentially hydrolyze sphingomyelin and unsaturated phosphatidylcholine to toxic compounds that damage the RBC membrane, resulting in intravascular hemolysis [107,120]. The toxin changes the physical properties and morphology of the erythrocyte membrane and, moreover, it impairs erythropoiesis by the inhibition of erythroid differentiation [121] and disturbing the production of RBCs. The RBC membrane is also target of a number of bacterial pore-forming cytolysins of the highy diverse RTX (repeats in toxin) family [122]. Their common feature is the release via the type I secretion system and the typical glycine- and aspartate-rich nonapeptide repeats that can bind a large number of Ca^2+^ ions [123]. The generation of pores by RTX toxins leads to the collapse of ion gradients and the membrane potential across the plasma membrane of target cells, which results in cell death [124]. A prototype member of the RTX pore-forming toxins is the α-hemolysin, which is often encoded by strains of uropathogenic *E. coli* (UPEC) [125]. It correlates with the strength of infection as the majority of UPEC isolates from pyelonephritis cases express α-hemolysin [126]. The enterohemolysin of EHEC, also termed EHEC-hemolysin (EHEC-Hly), is a further member of the RTX family regarded as a potential virulence factor frequently associated with severe human disease such as hemorrhagic colitis and HUS [127,128]. EHEC-Hly is a membrane pore-forming toxin and demonstrates similar efficiency in the lysis of sheep and human erythrocytes [129]. Upon entering the circulation, EHEC-Hly may cause RBC destruction and its activity, especially in the absence of neutralizing antibodies, may directly lead to hemolysis along with increased levels of intravascular heme [127,128]. Moreover, besides the formation of pores in human RBCs, EHEC-Hly was shown to induce the production of IL-1β from human monocytes, which is one of the serum risk markers for HUS [130]. Since it is known that IL-1β increases the biosynthesis of the Stx receptor globotriaosylceramide (Gb3Cer) of human endothelial cells [131], it is tempting to speculate about implications of EHEC-Hly for the pathogenesis of HUS by enhancing the detrimental effect of Stx. In addition, EHEC-Hly *per se* is capable of injuring human endothelial cells as shown for Stx-negative *E. coli* O26 strains isolated from patients with HUS [132] and to cause endothelial and epithelial apoptosis [133]. EHEC-Hly is secreted extracellularly both in a free soluble form and associated to outer membrane vesicles (OMVs) [134]. The OMV association stabilizes the RTX toxin and considerably prolongs its hemolytic activity compared to the free form [127]. Of note, recent findings suggest that OMVs provide a general concept for the stabilization of EHEC virulence factors, opening new insights into the mechanisms of cell interaction as well as the intracellular delivery, trafficking, and mechanisms of vesicularly stabilized toxins [135]. However, the primary and best characterized virulence factor of pathogenic *E. coli* is Stx of the AB_5_ family of protein toxins released by Stx-producing *E. coli* (STEC) [136,137,138,139,140,141]. Although Stx can bind to human erythrocytes [142,143], direct damage of the erythrocyte cell membrane caused by Stx has, to the best of our knowledge, never been reported. Interestingly, in this context, evidence has been provided that Stx is capable of injuring developing erythrocytes [144,145]. Experimental data showing the cytotoxic effects of Stx toward certain developmental stages of nucleated erythroblasts will be intensively described and discussed below in the main chapter of this review (see Section 9).

## 6. A Short Historical Reflection on Glycosphingolipids of Mature and Developing Erythrocytes

The next remarks start with a concise historical review on the detection and structural identification of globo-series GSLs of human erythrocytes, focusing on the eponymous GSLs Gb3Cer and Gb4Cer. Then, we shortly debate the GSL composition of human myeloid and lymphoid cells and close the chapter with an outline on the GSL expression of erythroleukemic cell lines, which are commonly used as models of erythrocyte differentiation in vitro.

### 6.1. Glycosphingolipids of Human Red Blood Cells

**Figure 2:**
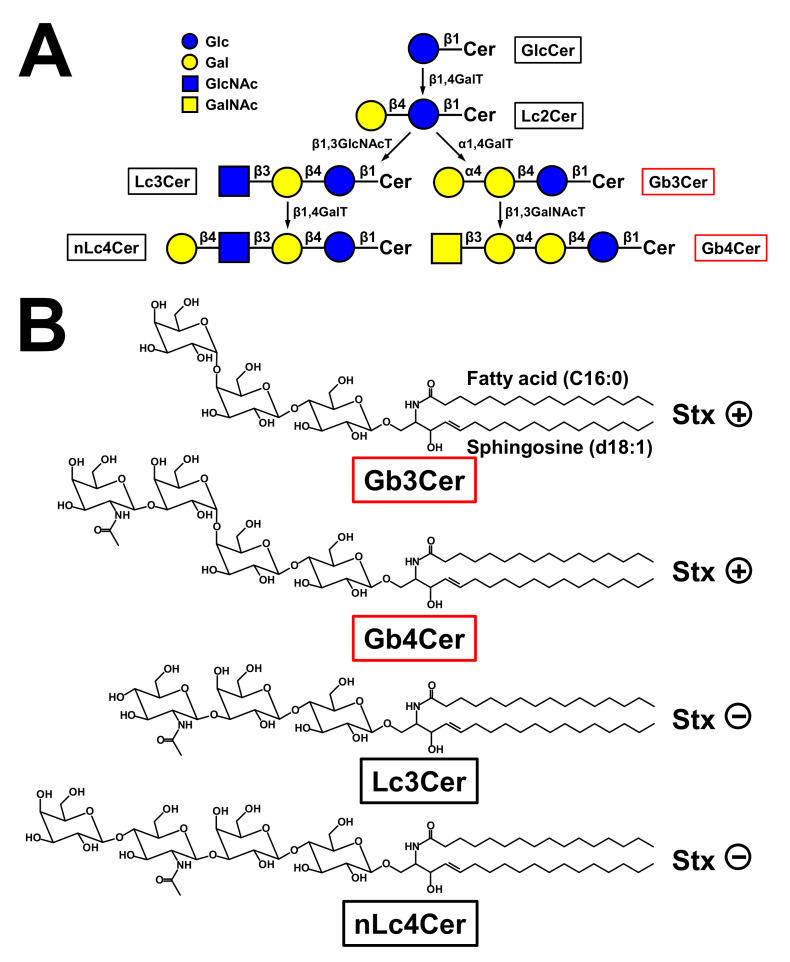
Biosynthesis flow diagram of globo-series Gb3Cer and globotetraosylceramide (Gb4Cer) and neolacto-series lactotriaosylceramide (Lc3Cer) and neolactotetraosylceramide (Lc4Cer) (**A**) together with the related structures and Shiga toxin (Stx)-recognition competence (**B**). (**A**) Galactose is transferred to glucosylceramide (GlcCer) by the action of a β1,4-galactosyltransferase (β1,4GalT) producing lactosylceramide (Lc2Cer), which represents the precursor globo-series glycosphingolipids (GSL) and linchpin for the biosynthesis of the various GSL families. Right side: Gb3Cer is the first globo-series GSL being produced by an α1,4-galactosyltransferase (α1,4GalT) that adds a galactose molecule in α1-4-configuration to Lc2Cer. Then, Gb4Cer is synthesized by action of a β1,3-*N*-acetylgalactosaminyltransferase (β1,3GalNAcT). Left side: Lc3Cer is produced by a β1,3-*N*-acetylglucosaminyltransferase (β1,3GlcNAcT) that adds an *N*-acetylglucosamine molecule in β1-3-configuration to Lc2Cer. Then, the neolacto-series GSL nLc4Cer is synthesized by action of a β1,4-galactosyltransferase (β1,4GalT). (**B**) The structures of Gb3Cer and Gb4Cer as well as Lc3Cer and nLc4Cer are depicted in the chair conformation. The four GSLs are exemplarily portrayed with a ceramide (Cer) moiety carrying sphingosine (d18:1) and a C16:0 fatty acid in the double-tailed Cer (d18:1, C16:0) lipid anchor. Both Gb3Cer and Gb4Cer are recognized by Stx (Stx**+**), whereas Lc3Cer and nLc4Cer are not (Stx−).

Two fundamental reviews published by Sen-itiroh Hakomori in 1981 and Minoru Fukuda in 1985 gave profound insights into the surface glycoconjugate structures of hematopoietic cells [146,147]. One important message taken from these reviews was the recognition that GSLs are frequently specific to the divergent cell lineages of the hematopoietic system and, in particular, to the developing and mature erythrocytes of the erythroid lineage. By considering the molecular structures of GSLs, they are basically composed of a hydrophilic oligosaccharide moiety and a twin-tailed hydrophobic ceramide (Cer) portion, built up from eponymous sphingosine (d18:1), a dihydroxylated and mono-unsaturated amino alcohol with a C18 alkyl chain, and a fatty acid with varying carbon chain length [148,149,150,151,152]. For a long time now, it has been known that human erythrocytes do express neutral GSLs of the globo-series [153,154,155,156]. The major GSL of the globo-series is the tetrahexosylceramide Gb4Cer [157,158,159,160], which has been termed “globoside” by Tamio Yamakawa in 1952 [161], and it is also known as human blood group P antigen [162,163]. In human RBCs, Gb4Cer is accompanied by the less abundant ceramidetrihexoside (CTH) denoted as Gb3Cer, which is also known as blood group P^k^ antigen [162,163] (for structures, see Figure 2). As a key feature among the different GSL families, Gb3Cer and Gb4Cer exhibit galactose in α1-4-configuration linked to lactosylceramide (Lc2Cer, Galβ1-4Glcβ1-1Cer), which is the common precursor of the mammalian GSL families [151,152,164]. The first committed step in the biosynthesis of globo-series GSLs is executed by the enzyme UDP-Gal:Lc2Cer α1,4-galactosyltransferase (α1,4GalT) [165]. Resulting structures are Galα1-4Galβ1-4Glcβ1-1Cer (Gb3Cer) carrying a terminally α1-4-linked Gal and GalNAcβ1-3Galα1-4Galβ1-4Glcβ1-1Cer (Gb4Cer) corresponding to GalNAc-elongated Gb3Cer harboring a subterminally α1-4-linked Gal molecule (Figure 2). For the general structural diversity of GSLs beyond the globo-series and GSL biosynthesis pathways in general as well as their multiple functions in cellular processes such as development and differentiation, the reader should refer to a number of excellent reviews addressing these topics [151,152,164,166,167,168,169]. The Gb3 and Gb4 oligosaccharides are not found as *O*- or *N*-glycans on mammalian glycoproteins. Thus, the Gb3 and Gb4 glycans are unique among all known oligosaccharides with respect to their exceptional existence as lipid-linked structures in GSLs of the globo-series.

### 6.2. Glycosphingolipids of Human Myeloid and Lymphoid Cells and Cell Lines

Considering shortly the GSLs of non-erythrocyte cells of human blood, the GSL content of human erythrocytes contrasts with that of granulocytes, which are characterized by the neolacto-series GSL neolactotetraosylceramide (nLc4Cer), the presence of minor lactotriaosylceramide (Lc3Cer), and the omission of globo-series GSLs [170,171] (for structures, see Figure 2). However, at the early stage of neutrophil differentiation, myeloblasts do express, in addition to neolacto-series GSLs, the globo-series GSLs Gb3Cer and Gb4Cer [119]. Human monocytes express GSLs of both, i.e., the globo- and the neolacto-series, whereas the heterogeneous population of human T and B lymphocytes express mainly globo-series GSLs with a higher content of GSLs in B cells [172,173,174,175,176]. Interestingly, pre-B cells contain neolacto-series GSLs, which change during B cell differentiation to globo-series GSLs [177]. Human leukemic cells or cell lines that are arrested at a specific stage of hematopoietic/erythropoietic development are widely used, aiming at the identification of presumptive stage-specific or lineage-specific marker GSLs. However, one should be aware that some observed characteristic compounds might be related to malignancy rather than differentiation. The human monocytic THP-1 cell line is such a cell line being widely used as an in vitro phagocytic cell model owing to similar cellular properties to monocyte-derived macrophages [178]. Undifferentiated THP-1 cells express Gb3Cer and Gb4Cer [179,180,181], which decrease upon macrophagic maturation concomitant with severely modified surface glycosylation [178,182]. Last but not least, GSLs of the globo-series are absent in the Jurkat cell line (T cell descendant) and the HL-60 cell line (granulocyte lineage) [181], whereas Raji cells (B cell descendant) were found to contain Gb3Cer as the major neutral GSL [180,181], whereby corresponding α1,4GalT activity was found to correlate with the GSL content of analyzed HL-60 and Raji cells [165].

### 6.3. Erythroid Character and Glycosphingolipid Expression of the Human Erythroleukemic K562 and HEL Cell Lines

The biochemical analysis of the GSL expression of developing human erythrocytes became possible after establishing immortal human erythroleukemic cell lines arrested at distinct stages of erythropoietic differentiation as an alternative to primary cells. The erythroid nature of K562 cells, which were initially regarded as myeloid cells [183], was verified by demonstrating the presence of GPA, which represents the major glycoprotein of RBCs [184,185,186]. This highly glycosylated membrane protein is known to be expressed exclusively on basophilic erythroblasts and on later erythropoietic stages, but not on immature erythroblasts (proerythroblasts) [187]. The erythroid character was afterwards confirmed by the detection of inducible hemoglobin synthesis [188]. Subsequent investigations of the GSL composition of the K562 cell line revealed Lc3Cer and nLc4Cer as the characteristic neutral GSLs of K562 cells [189], whereas Gb4Cer, the major neutral GSL of mature erythrocytes, and Gb3Cer were detected only in very low amounts [190] (for structures, see Figure 2). These striking differences between K562 cells and mature erythrocytes gave a first hint that GSLs may be useful development-associated markers of normal erythrocyte differentiation [191]. Another human erythroleukemic cell line exhibiting an erythroid character is HEL [192], showing very similar cell surface properties when compared to K562 cells and being a valuable complementary cellular model for studying erythroid-specific proteins [147,186,193,194,195]. However, in contrast to the K562 cell line, HEL cells can be also differentiated to macrophage-like cells, suggesting that HEL cells are developmentally arrested at an earlier erythropoietic stage than K562 cells [196]. Similar to K562 cells, HEL cells showed a remarkably lower content of globo-series GSLs [190]. Thus, both erythroleukemic cell lines, K562 and HEL, serve as classical models of erythroid differentiation in vitro and the acquisition of an erythroid phenotype upon exposure to appropriate inducers [193,197,198,199,200,201,202,203,204].

## 7. EHEC-Caused Diseases and Damage of Human Target Cells

Starting this chapter, we above all touch an evolutionary aspect on the primordial Stx-based defense mechanisms of STEC against protozoan predators in a “non-clinical environment”. Then, we highlight the current knowledge on the pathogenicity of Stxs released by EHEC strains. The third part considers the Stx structure and Stx-mediated cellular impairment effects, followed by a short compilation that delineates the Stx target cells residing in the human colon, kidneys, and the brain.

### 7.1. Shiga Toxin as Primordial Bacterial Weapon Against Eukaryotic Predators

From the perspective of evolution, bacterial pathogens may have acquired their pathogenic capability by incorporating genetic elements through horizontal gene transfer, whereby the ancestors of infectious bacteria most likely derive from natural ecosystems of the environmental microbiota [205]. For this reason, exotoxin-mediated killing of protists represents a basic principle of bacterial defense against unicellular eukaryotic predators. The unexpected high frequency of exotoxin-coding genes in regions lacking the presumed mammalian hosts suggest that (1) mammals are not their primary targets and (2) exotoxins such as Stx may have evolved for the purpose of bacterial antipredator defense [206,207]. The bacterivorous predator *Tetrahymena thermophila* is killed in cocultures with STEC for which the Stx-encoding bacteria enhance survival in the face of protist predation over those bacteria that are negative for Stx expression [208]. Bacteriophage-mediated lysis of Stx-encoding bacteria is required for Stx cytotoxicity in *Tetrahymena.* Thus, Stx must be released prior to digestion, since toxin released as a consequence of digestion is harmless to the protozoan [209]. Phage-encoded exotoxins including Stx kill mammalian cells by the impairment of universally conserved factors or pathways after internalization, although the existence of Gb3Cer or a Gb3Cer-analogous receptor has never been described for *Tetrahymena* species and remains obscure [209].

### 7.2. EHEC-Caused Life-Threatening Diseases

EHEC are zoonotic pathogens that are capable of causing deadly epidemics [210]. Ruminants are symptomless carriers of EHEC bacteria and are recognized as their primary natural reservoir [211,212]. Cattle represent the most important source of human infections, where EHEC localize in the recto-anal junction of the animals [213,214,215]. EHEC O157 outbreaks are mostly linked to the consumption of contaminated bovine-derived products, including animal contact in petting zoos with lower incidence, as sources of STEC infections [211,213,216,217,218,219]. After ingestion, EHEC selectively colonize the mucosa of the human large intestine with the “attaching and effacing” mechanism, genetically governed by a large pathogenicity island defined as the Locus of Enterocyte Effacement (LEE) [211,220,221,222,223,224,225]. Besides severe diarrhea and hemorrhagic colitis, EHEC raise life-threatening extraintestinal complications such HUS with frequent long-term and grave sequelae. These relate to hypertension, permanent residual kidney dysfunction, or persistent proteinuria with the risk of progressing to chronic renal failure and end-stage renal disease after more than 5 years, and sometimes as late as 20 years, after the acute disease [226,227,228,229,230,231,232,233,234,235]. Extrarenal complications in Stx-mediated HUS affecting other organ systems including the central nervous, gastrointestinal, cardiac, and musculoskeletal systems have been reported as well, and they do occur not only in the acute setting but may also be seen well after recovery from the acute phase of HUS [236,237]. HUS is characterized by the simultaneous occurrence of hemolytic anemia (anemia caused by the destruction of erythrocytes), thrombocytopenia (low platelet count), and acute kidney failure (uremia) [227,238,239,240,241], while damage of the brain results in serious neurological disorders [233,236,237,242,243,244]. Neurological injury can be sudden and severe and is the most frequent cause of acute mortality in patients suffering from vigorous EHEC infections [233,245]. Of note, EHEC-derived Stxs are also capable of activating multiple cell stress signaling pathways, which may converge to innate immune responses and inflammation, thereby increasing the severity of organ injury in infected patients [246,247,248,249]. This is further aggravated by the fact that Stx interacts with the complement system, resulting in enhanced complement activation [250,251,252]. To date, there is no specific therapy for EHEC-associated HUS, but patients benefit from supportive care [253,254]. In particular, antibiotic treatment is controversial and a matter of debate, because at least some antibiotics may increase the risk of HUS [255,256,257,258,259,260,261]. In this context, it might be of interest that lower erythrocyte Gb3Cer levels in comparison to healthy controls were found to associate with HUS, showing an interesting relationship between differential susceptibility to HUS and erythrocyte Gb3Cer content [262]. Such an altered Gb3Cer profile might eventually reflect a genetic predisposition for the differential outcome of EHEC infections.

EHEC of various serotypes release Stxs as their major virulence factors, whereby Stx1a and Stx2a (in previous publications imprecisely denoted as Stx1 and Stx2, respectively) are the clinically most relevant subtypes for humans [139,227,263,264]. Stxs are presently the best characterized virulence determinants of EHEC strains being differently associated with the risk of developing severe course of the disease [265,266,267,268]. Epidemiologically, Stx2a seems to be more important than Stx1a in the development of HUS [136]. The globally widespread EHEC of serotype O157:H7 is responsible for most STEC infections [227,269,270,271]. Among the numerous non-O157 serogroups associated with outbreaks and sporadic illness, the serogroups O26, O45, O103, O111, O121, and O145 have been reported in the past to account for the vast majority of reported non-O157 STEC infections worldwide [218,272,273,274,275]. However, the devastating 2011 outbreak in Germany was caused by the “unusual” EHEC serotype O104:H4 [217,259,276,277] and has been portrayed by 855 HUS cases and 53 deaths [278,279]. A subsequent experimental infection study of calves with the outbreak strain provided first evidence that cattle can be colonized by unusual EHEC strains such as O104:H4 [280].

### 7.3. Shiga Toxin and Toxin-Mediated Cell Damage

The ensuing chapters are dealing first of all with a short description of the classical AB_5_ structure of Stx and the *N*-glycosidase-mediated depurination of certain adenosines of ribosomal RNA and nuclear DNA caused by the catalytically active A1 fragment. Subsequently, the Stx binding specificity of the B pentamer is elucidated, followed by a short survey of the remarkable interaction of the toxin’s A subunit with Toll-like receptor 4 (TLR4) and the intracellular retrograde routing of Stx.

#### 7.3.1. Structure of Stx and Enzymatical Depurination of Ribosomal RNA and Nuclear DNA

All Stxs share an AB_5_ structure, built up from a single A subunit non-covalently linked to five identical B subunits [140,281,282,283,284] similar to the subtilase cytotoxin (SubAB), which represents the prototype of a “new” family of potent AB_5_ cytotoxins produced by STEC strains [285,286,287,288]. The 32 kDa A subunit of Stx is made of a large enzymatically active 27.5 kDa A1 and a small 4.5 kDa A2 fragment, which are linked via a disulfide bond [136]. The B pentamer consists of five identical 7.7 kDa B subunits forming a doughnut-shaped structure that surrounds the A subunit near the C-terminus [289]. Crystallographic studies have shown that the active site of the A1 fragment of Stx2a from *E. coli* O157:H7 binds to a specific adenosine of the ribosomal RNA underlining the toxin’s *N*-glycosidase activity [290,291]. In this context, we highly recommend the expert review recently published by Chan and Ng [140] dealing with latest Stx-related topics and tracing an arc from the structure and mechanisms to applications of Stxs.

Stxs belong to the type 2 (two-chain) ribosome-inactivating proteins (RIPs) [138,292,293,294,295]. The unique *N*-glycosidase activity of the A1 fragment targets not only a universally conserved adenosine in the α-sarcin loop of the 28S ribosomal RNA of the eukaryotic 60S ribosomal subunit, but it also depurinates adenosines of various polynucleotide substrates and nuclear DNA, leading to lesions of the cell nucleus [296,297,298,299,300]. The enzymatic inactivation of eukaryotic ribosomes results in the irreversible abrogation of cellular protein biosynthesis and, thus, leads to ultimate cell death [301]. Moreover, many studies suggest that Stx induces apoptosis in endothelial, epithelial, and other cell types [141,302,303] and are capable for eliciting a ribotoxic stress response [246,304,305] again confirming Stxs as multifunctional proteins [247].

#### 7.3.2. Stx Binding Specificity of the Inherent B Pentamer

Stx is a member of the group of galactose-specific RIPs comparable to the heterodimeric highly toxic AB plant protein ricin, which is produced by the seeds of the castor oil plant *Ricinus communis* [306,307]. The B subunit of ricin binds to glycans bearing β1-4-linked galactose residues [306] with the preference of Galβ1-4GlcNAc > Galβ1-3GalNAc > Galβ1-4Glc as determined with GSLs harboring the mentioned structures with terminally β-configurated galactose molecules [308]. In contrast, the B pentamer of Stx binds to globo-series GSLs [309] exhibiting a keen preference for the Gb3Cer GSL unique for the Galα1-4Galβ1-4Glc trisaccharide [137,139,284,310,311,312,313]. This holds true for the human–pathogenic subtypes Stx1a and Stx2a, which recognize also Gb4Cer but to a lesser extent than Gb3Cer [314,315,316,317]. Remarkably, the swine-pathogenic Stx2e is special among the various Stx subtypes showing, besides binding toward Gb3Cer, a pronounced preference toward Gb4Cer carrying the GalNAcβ1–3Galα1–4Galβ1–4Glc tetrasaccharide [316,318,319] and a promiscuous binding activity toward elongated Gb4Cer structures. These are globopentaosylceramide (Gb5Cer) with Galβ1–3GalNAcβ1–3Galα1–4Galβ1–4Glcβ1–1Cer structure [320] and GalNAcα1–3GalNAcβ1–3Galα1–4Galβ1–4Glcβ1–1Cer, which is defined as the Forssman GSL [316,321]. A Gb3 analogue trisaccharide was found to bind to the 3 densely located binding sites of each of the identical B subunits, whereby all 15 trisaccharide molecules bind to one side of the B pentamer, indicating that this side faces the cell membrane [290,291,322,323,324]. Although binding site 2 was the key site in terms of binding using free glycans, site 2 alone is not sufficient to confer high avidity attachment to membrane-localized Gb3Cer. Furthermore, the membrane environment was found to be essential for biologically relevant studies of the interaction based on investigations using Gb3-decorated liposomal membranes [323].

#### 7.3.3. Interaction of the A Subunit of Stx with the Toll-like Receptor 4

An alternative non-GSL receptor has been detected for Stx, based on early findings that Stx sticks toward human granulocytes [325]. Granulocytes (see Figure 1) do not own globo-series Gb3Cer and Gb4Cer, which are the well-known receptor GSLs for Stxs, but they have the neolacto-series GSLs Lc3Cer and nLc4Cer [170,171], which do not bind to Stxs (for structures, see Figure 2). The strength of Stx adhesion to granulocytes was 100-fold less than that of Stx toward Gb3Cer. This rather low binding affinity allows the transfer of Stx from Stx-preloaded granulocytes to human glomerular microvascular endothelial cells, which do express the high-affinity receptor Gb3Cer being recognized be the B pentamer of Stx [325]. Hereafter, Stx-carrying granulocytes were detected in the systemic circulation of children suffering from HUS [326,327]. Furthermore, Stxs were detectable for a median period of 5 days providing a valuable tool for the laboratory diagnosis of STEC infection in HUS [328]. The role of granulocytes as carriers for Stx was scrutinized in a study that showed the passage of Stx from older granulocytes to new, mature cells entering the circulation from the bone marrow [329] and explained the previously reported persistence of Stx in the blood of children with HUS [328]. Investigations aimed at identifying the non-GSL receptor of Stx on human granulocytes [330] finally yielded TLR4 as the receptor in human neutrophilic granulocytes that recognizes Stxs [331], in contrast to human monocytes, where Stx interacts via Gb3Cer in terms of releasing HUS-associated proinflammatory mediators [332]. Of note, the antibiotic polymyxin B is capable of impairing the interaction between Stx and human neutrophilic granulocytes [333] and, moreover, the soluble extracellular domain of TLR4 was found to inhibit the adhesion of Stx to neutrophilic granulocytes [334]. Stx2a complexed with soluble TLR4 escaped from capture by human serum amyloid P component (HuSAP), allowing the toxin to target and damage human cells. HuSAP is considered a negative modulating factor that specifically binds Stx2a and abrogates its toxic action, suggesting soluble TLR4 as a positive modulating factor for Stx2a [334]. Collectively, the interplay of Stx with TLR4 suggests a protein–protein interaction mechanism between the Stx A subunit and TLR4 that seems to be independent from the protein–carbohydrate interaction between the Stx B pentamer and Gb3Cer.

#### 7.3.4. Retrograde Transport of Stx

Upon receptor-mediated binding of the pentameric B subunit to cell surface-exposed Gb3Cer, Stx enters an intracellular retrograde trafficking route from the plasma membrane through the Golgi network to the endoplasmic reticulum, followed by cleavage of the A subunit and translocation of the catalytically active A1 fragment into the cytosol, where it exerts its cytotoxic action [138,140,141,283,301,335,336]. The Stx-binding GSLs are not randomly distributed in the plasma membrane, but they are organized in liquid-ordered nanometer-sized clusters as dynamic microdomains denoted as lipid rafts. They float freely in the membrane bilayer [337,338,339], thereby interacting with actin-connecting proteins and the underlying cytoskeleton, regulating many facets of eukaryotic cell function [340,341,342]. Recent findings suggest an interdigitation between “very-long-chain” (glyco)sphingolipids of the outer membrane leaflet and phosphatidylserine (18:0/18:1) in the inner membrane leaflet, which are termed as “handshaking” of the two partners. It can be speculated that such interleaflet coupling between the “very-long-chain” Gb3Cer (d18:1, C24:0/C24:1) and phosphatidylserine (18:0, 18:1) in conjunction with cholesterol may play an important role for the intracellular signaling of Stx [343,344,345]. GSLs are closely associated with cholesterol AD sphingomyelin, which rank among canonical lipid raft markers, and membrane proteins interacting with these classes of lipids [346,347,348,349]. Attachment, uptake, and endocytosis of Stx and related AB_5_ toxins may occur most efficiently when the GSL receptors are inserted in lipid rafts [350,351,352,353,354], which is a process that is excluded, for instance, under conditions of cholesterol depletion [350,355]. The clustered occurrence of Stx-binding GSLs in human renal glomeruli may define a glomerular- and age-restricted pathology of Stx-caused HUS and has been hypothesized as the first example, where membrane Gb3Cer organization may predict a tissue selective in vivo pathology [356,357,358]. Moreover, Stx-induced tubular membrane invaginations were discovered as a new principle for Stx uptake into cells providing a rationale for the various endocytic uptake processes and the bewildering diversity of endocytic routing of the Stx–GSL complex to the cell interior [359,360,361,362]. It is hypothesized that lipid rafts are the origin of vesicular trafficking [339] and that additional factors such as the density of Gb3Cer in lipid rafts may have an effect on binding [363] and that the co-assembly with other GSLs may influence the extent of Stx-mediated cellular damage [352].

### 7.4. Human Target Cells of Shiga Toxins

The emphasis in the following paragraphs was put on the interplay of Stx with the human intestinal epithelium and EHEC-released Stx-carrying outer membrane vesicles in the intestine as well as Stx-mediated extraintestinal complications after transfer into the circulation such as HUS and cerebral dysfunction. In this context, an Stx shuttle by cellular compounds and Stx-loaded microvesicles play a pivotal role targeting not only endothelial cells, but also epithelial cells and other cells of the kidney, rounding off the topic of this paragraph.

#### 7.4.1. Interaction of Stx with the Human Intestinal Epithelium

The exact mechanism of how Stx attaches to the human intestinal epithelium, crosses this cellular barrier, and gains access to the blood stream is a matter of debate and remains in a number of ways enigmatic [221]. Macropinocytosis and the transcytosis of Stx across intact intestinal epithelial cells are steps that are necessary for its systemic spread, without apparent cellular damage having been demonstrated for Stx1 using a cell culture electrical resistance in vitro model employing the human CaCo2A and T84 colon cancer cell lines [364,365]. On the other hand, Stx1 and Stx2 were found to cause the inhibition of protein synthesis and apoptosis in Gb3Cer-positive Caco-2 cells but not in Gb3Cer-negative T84 cells [366]. Of note, both Stxs were internalized and directed to the endoplasmic reticulum in both cell lines, indicating a Gb3Cer-independent transport route in T84 cells for Stx that does not induce cell damage in the Gb3Cer-deficient cell line [366]. However, the expression of Gb3Cer in metastatic colon cancer cells and cancer-derived cell lines such as the Caco-2 [367,368] versus postulated absence in normal human epithelial cells of the small intestine [369] and the large intestine [370] suggests its association in metastatic transformation among a colon tumor cell population [371]. Unlike the general assumption, the presence of Gb3Cer has been indirectly shown by the binding of Stx1 and Stx2 toward colonic epithelia in fresh human tissue sections along with the detection of Gb3Cer synthase mRNA [372]. This finding was further supported by the presence of the lower-affinity Stx receptor Gb4Cer, suggesting that Gb3Cer may exist in small quantities in human colonic epithelia, where it may compete for Stx binding with more abundant Gb4Cer [372]. Simulation of the microaerobic environment in the human intestine and the application of a vertical diffusion chamber using T84 colon carcinoma cells provided novel insights into alternative virulence strategies of Stx-producing *E. coli* O157:H7 and O104:H4 [373]. The authors could show a significantly reduced bacterial growth as well as a decreased production and release of Stx at microaerobiosis, whereas translocation across the epithelial cell layer was enhanced under microaerobic versus aerobic conditions, suggesting that the microenvironment in the human colon may modulate Stx-related events and enhance the absorption during STEC infection [373]. Importantly, the T84 microaerobic infection model revealed evidence for substantially lowered Stx2a translocation across the colon epithelial cell layer in STEC strains rarely or not linked to human disease compared to STEC strains associated with severe human intestinal disease and outbreaks [374]. Thus, high Stx2a translocation efficacy correlates with the strong virulence of Stx-producing *E. coli*, arguing that the extent of Stx transcytosis across the intestinal epithelium may represent an important indicator of STEC pathogenicity for humans [374]. Last, but not least, Stx has been shown to elicit a ribotoxic stress response via the stimulation of classical mitogen-activated protein kinases in the colorectal carcinoma cell line HCT-8, which is known to harbor Stx-binding GSLs of the globo-series [368], thereby contributing to Stx-induced inflammation [375]. Collectively, although a number of studies have provided evidence of possible Stx-mediated damage of the human colon epithelium, the data are chiefly based on in vitro cell culture models utilizing human colon carcinoma cell lines, which do not reflect the in vivo conditions. Thus, the existence, for instance, of Gb3Cer or Gb4Cer in cancer-derived cell lines cannot be taken as a proof for their existence in normal colon epithelium, since the molecular pattern of GSLs may change in cancer cells with respect to the healthy counterpart. Thus, exploring the in vivo situation of Stx-mediated injury and unraveling the mechanism employed by the toxin to pass from the intestinal lumen to underlying tissue and to enter the systemic circulation still remains a challenging approach for future research. Potential routes are a Gb3Cer-mediated translocation by Paneth cells, a paracellular “piggy-back” transport through neutrophil transmigration or transcytosis by M cells, thus pointing to a few knowledge gaps in our understanding of the early event of STEC infection. The causal mechanisms of this yet understudied field are far from being clarified and need to be addressed further [221].

#### 7.4.2. EHEC and Outer Membrane Vesicles

In the human colon, Stxs may be released by EHEC in free form through phage-induced bacterial cell lysis by decaying bacteria, since no specific secretion system has been identified so far for the active release of Stxs [140,221,257]. Noteworthy, liberated Stx phages can infect not only *E. coli* but also other types of bacteria, such as *Citrobacter freundii* or *Enterobacter cloacae*, and may “abuse” susceptible bacteria in the population as surrogates to multiply toxin and phage production [376,377,378,379]. Thus, Stx-encoding bacteriophages have to be considered extremely mobile genetic elements that play a pivotal role in the (1) expression of Stx, (2) horizontal gene transfer, and more generally (3) genome diversification acting as “genomes in motion”, thereby strengthening the severity of STEC infections as prophesized by the Karch research consortium in 2004 [380]. As an alternative to release in free form, Stx was found entrapped in or associated with OMVs being shed from STEC during growth in vitro and in vivo. Thus, OMVs, composed of bacterial outer membrane wrapped around the contents of the periplasmic space, the inner membrane and the cytosol have been identified as a novel principle for interspecies communication of an increasing number of intestinal bacteria with host intestinal epithelial cells and an economic delivery strategy for the release of toxins [127,381,382,383]. First considered as a by-product of cell lysis, it soon became evident that these spherical nanostructures are actively shed from Gram-negative bacteria, thereby attracting attention as a highly conserved mechanism in the context of host–pathogen interaction and virulence regulation [133,384,385,386,387]. Evidence for this hypothesis with special reference to interrelationship of Stx delivery with OMVs has been provided by bacterial cell cultures of EHEC O157:H7 and O104:H4. Investigations on these outbreak strains producing Stx2a as the major virulence factor (besides others) indicated virulence from OMVs as an effective strategy of Stx-mediated host cell injury [135,261,388,389]. Thus, novel mechanisms of releasing a myriad of virulence factors, including Stx attached to or entrapped in OMVs derived from the EHEC outer membrane, represent unprecedented ways for EHEC strains to deliver pathogenic cargoes and harm host cells.

#### 7.4.3. Stx-Mediated HUS and Cerebral Dysfunction

Upon transfer into the circulation, Stx evokes life-threatening systemic extraintestinal complications such as HUS with a risk for the development of long-term chronic sequelae [226]. HUS is the leading cause of Stx-mediated kidney injury characterized by microangiopathic hemolytic anemia, thrombocytopenia, and acute renal failure [232,253,302,390,391,392]. The renal histopathology is characterized primarily by glomerular thrombotic microangiopathy with glomeruli showing morphological changes of the arterial and capillary endothelial cells and narrowing of the microvascular lumen [393]. More precisely, the term “thrombotic microangiopathy” defines a lesion of microvessel wall thickening, intraluminal platelet thrombosis, partial or complete obstruction of the vessel lumen, and associated organ dysfunction [394,395,396]. Hence, severe glomerular thrombotic microangiopathy with changes ranging from endothelial cell damage to overt thrombosis suggests that Stx-induced injury of renal microvascular endothelial cells of the glomeruli has been recognized as the trigger event of acute renal impairment that underlies the pathological changes in HUS [231,263,393,397]. Platelet activation leads to thrombocytopenia and vessel occlusion during HUS and is the result of platelet consumption in platelet–fibrin aggregates [398,399]. Plausible explanations for thrombus formation are the contact of Gb3Cer-containing platelets with aggregating agents such as Stx known to bind to and activate platelets [400,401] or binding to the surface of Stx-injured endothelium. Activated endothelial cells in response to Stxs lose the normal thromboresistance phenotype and become thrombogenic, initiating microvascular thrombus formation [231]. Stxs induce the expression of adhesive molecules culminating in leukocyte adhesion and platelet thrombus formation and, together with complement activation, confer the glomerular endothelium a thrombogenic phenotype [252]. In addition, cerebral microvascular endothelial cells are targeted by Stxs, leading to injured brain with the associated endothelial dysfunction considered responsible for neurological complications [140,233,234,236,395]. Disturbance of the endothelial blood–brain barrier elicits serious cerebral malfunction and neurological complications comprising an array of symptoms of the central nervous system such as altered mental status, seizures, stroke, and coma [237,242,243,302]. Collectively, although thrombotic microangiopathy mainly affects the microvasculature of the kidneys, vascular beds of other organs are affected as well, and the net result is a multi-organ thrombotic process [254,397,402]. A hallmark of thrombotic microangiopathy is the mechanical fragmentation of erythrocytes due to increased vascular stress by the microvascular thrombi, which is a setting event that may then sustain and amplify the microangiopathic process, resulting in hemolytic anemia and hemolysis [394,396].

#### 7.4.4. Cellular Stx Shuttle in the Bloodstream and Microvesicles

Once entered into the circulation, Stx is disseminated through the bloodstream and delivered to the principal target cells in the human body videlicet microvascular endothelial cells of the kidneys and the brain. This shuttle happens most likely by cellular blood components and/or macromolecular assemblies, suggesting several processes operating independently from each other. In the blood, neutrophilic granulocytes are considered transport vehicles of the toxin cargo through circulation [325,326]. The presence of Stxs on granulocytes circulating in the blood of children with HUS and correlation with Stx amounts in the intestinal lumen of the patients was shown by the Brigotti group [328]. The binding of Stx was corroborated in a subsequent investigation of the same group, showing that Stx-coated granulocytes are capable of transmigrating through confluent monolayers of endothelial cells and to transfer Stx to the target cells, resulting in significant cellular damage [403]. Blood cells, cellular aggregates, or cellular compounds carrying the high-affinity receptor Gb3Cer may thus, according to the given explanations above in the context of Stx A, subunit interaction with granulocyte TLR4 (see Section 7.3.3), being excluded from shuttling Stxs through the circulation and transfer to target cells. These are monocytes [181,404,405], leukocyte-platelet aggregates [406], or platelets [400,401]. For further details, the interested reader should refer to a nice review of Brigotti released in 2012 covering the proposed Stx carriers in the bloodstream and their role in renal damage in overt EHEC-caused HUS [407]. Further candidates that might act as macromolecular shuttle vehicles in the human bloodstream are plasma lipoproteins deduced from their content of Stx1a- and Stx2a-binding Gb3Cer [408]. However, this assumption to serve as possible Stx transport and transfer molecules has not yet been verified. As a novel mechanism of how bacterial virulence factors may gain access to the circulation and thereafter cause organ damage, the transfer of Stx entrapped within host blood cell-derived microvesicles has been reported by the Karpman group [409]. The researchers could show that blood cell-derived microvesicles harboring Stx were endocytosed by in vitro cultivated human renal endothelial cells, leading to the shutdown of protein biosynthesis and ultimate cell death, supporting the idea of a novel virulence mechanism in which the toxin can beyond that evade the immune system [409]. Microvesicles belong to the group of extracellular vesicles including exosomes and apoptotic bodies that are small membranous beads ranging from 30 nm to 5 µm in size [344]. They are shed by cells during activation, injury, and/or apoptosis, carrying components of parental cells and enable cells to rid themselves of unwanted substances [410,411]. With reference to EHEC infections, the involvement of blood cell-derived microvesicles in all categorical aspects of Stx-mediated hemolysis and Stx-associated HUS, thrombosis, and renal feature has been summarized in a readable review [412]. In a very recent study, “particulate” Stx, i.e., Stx entrapped in microvesicles (vesicular Stx), was shown being associated with the development of HUS in children [413]. Importantly, the distinctive feature of the patients who developed HUS (compared to those who recovered) was the presence of vesicular Stx2 in blood the day before diagnosis of HUS, suggesting the involvement of vesicular, blood cell-derived Stx2 in the transition from hemorrhagic colitis to HUS [413]. However, mechanical stress in the course of thrombotic microangiopathy, caused by vessel wall thickening, intraluminal platelet thrombosis, and partial or complete obstruction of the vessel lumen, may provoke the shedding of erythrocyte membrane vesicles, which is known as “blebbing” [414], “vesiculation” [415], and “fragmentation” of RBCs [416], resulting in hemolysis and hemolytic anemia as characteristic features in the onset of HUS [394,396]. Thus, vesicles as remnants of mechanical RBC membrane disrupture due to increased shear forces in the microangiopathic process constitute a further resource of vesicular Stx carriers. The same holds true for membrane fragments derived from eryptotic blebbing events (see Section 3.3), considering such erythrocyte remnants as potential Stx shuttle vehicles as well. Overall, Stx-loaded cellular blood components, namely granulocytes, and Stx-carrying microvesicles, released from various blood cells, may act in a multifaceted process to disseminate its toxic cargo through the circulation and to deliver it to endothelial cells of various vascular beds, preferably in the kidneys and the brain.

#### 7.4.5. Interaction of Stx with Non-Endothelial Cells of the Kidney

There is an increasing body of evidence that Stx may directly attack not only renal and cerebral endothelial cells leading to pathological malfunction of the endothelium that faces Stx-loaded granulocytes and/or Stx-carrying microvesicles. In terms of the kidney, Stx also damages other renal cells videlicet glomerular and tubular epithelial cells as well as mesangial cells [136,140,390,417,418]. To briefly explain these different cell types of the Bowman capsule, glomerular epithelial cells surround the glomerular capillary tuft as an envelope would, while tubular epithelial cells line the renal tubuli being connected with the renal capsule wall, and mesangial cells constitute the central stalk of the glomerulus [419,420]. Various Stx-mediated cell-damaging effects have been shown for normal human kidney (tubular) epithelial cells such as apoptotic cell death, the arrest of protein synthesis, a decrease in cell viability, an increase of Stx responsiveness by inflammatory factors, inhibition of water absorption, and negative impact on the cellular regeneration in 3D cultures [421,422,423,424,425,426,427,428,429,430]. Besides renal epithelial cells, diverse biologic responses were detected in human mesangial cells upon exposure to Stx such as the inhibition of protein synthesis, decrease in cell viability, reduction in nitric oxide production, and TNF-α-induced sensitization by Gb3Cer upregulation [335,422,431,432]. Direct tubular damage in vivo has been shown for Stx2 in a mouse model, suggesting the involvement of renal tubular epithelial cells in Stx-mediated kidney failure [433]. These reports indicate that, in addition to renal endothelial cells, a variety of non-endothelial cell types, such as epithelial and mesangial cells of the kidney, has so far been confirmed as direct targets for Stxs. Thus, glomerular pathology in HUS may also result from cumulative effects of Stx on non-endothelial cells, contributing to the aggravation of the thrombotic microangiopathy and renal failure in HUS.

## 8. Erythrocyte Morphology in the Microcirculation and Hemolysis

In this chapter, we highlight the flexible shape of erythrocytes, which can be precisely studied these days with novel microfluidic models aimed at analyzing microcirculatory dynamics. Particularly, the capability of erythrocytes to unscathedly pass through narrow microvessels is addressed. In this regard, we critically scrutinize the common and easily traceable opinion that vascular occlusion in the course of the development of HUS may lead to the mechanical disruption of erythrocytes and hence to intravascular hemolysis and ultimate hemolytic anemia.

### 8.1. Blood: A Juice of Very Special Kind

In Goethe’s *Faust*, Mephistoteles stated „Blood is a juice of very special kind“, laying emphasis on blood as the essence of life being nowadays an indispensable means for clinically required blood transfusion [434]. More specifically, blood is a two-phase suspension of formed elements (erythrocytes, leukocytes, and platelets) dispersed in an aqueous solution of organic molecules, proteins, and salts called plasma [435]. Erythrocytes are nucleus-free, devoid of DNA and RNA, apparently unable to synthesize proteins, and consequently have limited repair capabilities. RBCs are biconcavely shaped discs consisting of a lipid bilayer with an attached spectrin-based cytoskeleton. Consequently, they are remarkably flexible and deformable based on the spectrin network that endows the RBCs with shear elasticity [436]. The motion of RBCs in the microcirculation plays a central role in blood flow resistance and cell partitioning within the microvasculature. Erythrocytes can readily change their shape when exposed to mechanical forces in the bloodstream and can flow smoothly without any damage when passing narrow capillaries, which is a feature that can be significantly altered under pathological conditions. Narrow capillaries determine the erythrocytes’ flow-induced morphological alterations including the change of the biconcave discoid shape to parachute and slipper shapes observed in microchannels, which serve as idealized microvessels [437,438,439,440,441]. Improvements in experimental technologies using microfluidic models allows for the exact determination of applied shear stress and associated forces toward RBCs, their microcirculatory dynamics, mechanical stability and deformability, heterogeneity in rheological properties, the deformation of molecular architecture as well as hydrodynamic and macromolecule-induced interaction [436,442,443,444,445,446,447,448]. The specific mechanical and hemodynamic properties of RBCs contribute to aiding blood flow especially when exposed to shear forces in the microcirculation that may lead to morphological changes and associated vesicle formation [435]. For instance, microfluidic tools enable scientists to create physiologically relevant culture models taking advantage of the small dimensions resembling many features of the in vivo vascular microenvironment with fine spatial and temporal resolution excellently reviewed by Wong and co-workers [443]. The visualization of cytoskeleton-induced protrusions on RBC surfaces performed by computer simulation revealed that membrane blebbing can be elicited when the cytoskeleton is subject to a localized ablation or a uniform compression [414] and that the vesiculation of mature RBCs contributes to the removal of defective patches of the erythrocytes membrane [415]. However, it is important to point out that the extreme deformability allows RBCs to squeeze through occluded capillaries without any damage [436].

### 8.2. Microangiopathy and Hemolytic Anemia

Thrombotic microangiopathy due to Stx-induced endothelial functional disorder represents the clinical picture of thrombocytopenia and hemolytic anemia in the setting of small blood vessel thrombosis in the course of developing HUS [231,399,449]. These are the hallmarks in the pathogenesis of HUS caused by Stx-producing *E. coli* strains, and it is generally supposed that the mechanical disruption of RBCs evoked by increased shear stress in occluded microvessels results in intravascular hemolysis, a lowered number of RBCs, and ultimate microangiopathic hemolytic anemia [228,390,394,396,450]. HUS-associated symptoms such as abnormalities in erythrocyte morphology and erythroid fragmentation are attributed to enhanced shear forces to erythrocytes that have to squeeze through constricted vessels of the microvasculature as a consequence of the formation of microthrombi. So far, the possible involvement of Stx-damaged erythrocyte progenitor cells that may cumulatively contribute to the unfolding of mechanical load-induced intravascular hemolysis and hereinafter hemolytic anemia has to this day been largely ignored by the medical and scientific community.

## 9. Direct Shiga Toxin-Mediated Injury of Developing Human Erythrocytes

In the course of ex vivo purging of Stx receptor-expressing malignant cells from autologous stem cell grafts, a multifold depletion of tumor cells was achieved by making use of Stx’s cytotoxicity toward Gb3Cer-expressing malign cells, whereby erythrocyte progenitor cells escaped Stx cytotoxicity [451] (see scheme of hematopoiesis in Figure 1 as well as Section 2 and Section 3). This refractiveness rests on the absence of Stx receptors on human CD34^+^ hematopoietic progenitor cells, allowing the ex vivo use of Stx in purging Stx receptor-expressing malignant cells from autologous stem cell grafts [451]. However, the existence of Gb3Cer on myeloblasts, and possibly myeloid stem cells, representing early stages of myeloid differentiation, may have a remarkable impact on the application of Stx as an ex vivo purging agent [119]. Regarding the sensitivity of maturing erythrocytes, the exposure of erythroid cells in human cord blood cultures toward a combination of Stx and interferon-α had a direct toxic effect on the nucleated erythrocyte precursors [144]. On the other hand, Stx treatment without interferon-α, which has been shown to increase the biosynthesis of Stx-binding GSLs, had no significant effect on erythrocyte development. This study provided preliminary data on a synergistic effect of interferon-α and Stx on erythropoiesis, i.e., a direct and specific targeting of Stx onto developing erythrocytes, suggesting clinical relevance to infection with Stx-producing *E. coli* strains.

The following sections explain the current knowledge on the principles of ex vivo propagation of human CD34^+^ HSPCs, recently obtained data about the Stx2a-mediated damage of developing erythrocytes, their endowment with globo-series GSLs, and, finally, their Stx2a-mediated damage along with solid phase binding assays, demonstrating the attachment of Stx2a toward Gb3Cer and Gb4Cer isolated from ex vivo propagated erythropoietic cells.

### 9.1. Ex Vivo Amplification and Maturation of CD34^+^ Hematopoietic Stem/Progenitor Cells

In the chapters that follow, we start with the acquaintance about the clinical use of CD34^+^ HSPCs and the contemporary know how of stem cell mobilization, continue with the presentation of recent results on cytokine-induced ex vivo expansion and differentiation of erythroid cells as well as their concomitant morphological alterations, and finalize the section with a synopsis of morphological shifts recognized during ex vivo erythropoiesis.

#### 9.1.1. CD34^+^ HSPCs and Stem Cell Mobilization

As already explained above (see Section 2 and Section 3), erythropoiesis is a complex multistep process as part of the hematopoietic system (see Figure 1). An early step of maturing RBCs in the bone marrow niche is the genesis of multipotent CD34^+^ HSPCs, which subsequently traverse various intermediary developmental stages and end up in enucleated spheroid reticulocytes. At this late maturation stage, reticulocytes enter the bloodstream and mature in the blood to erythrocytes with characteristic biconcave shape (see Section 3 and Section 8). CD34^+^ stem cells have traditionally been used clinically to reconstitute the hematopoietic system (see Figure 1) after radiation or chemotherapy of a wide variety of malignancies [452]. CD34 represents an unambiguous molecular marker of hematopoietic stem cells that can be applied to the enrichment of HSPCs, because the pool of stem cells in vivo and in vitro consists of a mix of cells at several stages of differentiation that makes it difficult to attain a homogenous cell population [453,454]. Thus, CD34 has an enormous clinical utility in the identification, enumeration, and purification of engraftable hematopoietic progenitors for transplantation [452]. For this reason, several strategies for stem cell mobilization, i.e., the movement of stem cells from the bone marrow into the blood aimed at collecting a sufficient number of peripheral blood stem cells in response to stimulating factors, and harvesting techniques have been established since the early 1990s [455,456]. Most mobilization regimens are based on application by one or more growth factors such as granulocyte colony-stimulating factor (G-CSF) or granulocyte–macrophage colony-stimulating factor (GM-CSF), which are the standard agents approved for peripheral blood stem cell mobilization, or SCF, EPO, and other chemokines that synergize with G-CSF as a basis to promote alternate regimens and improve mobilization protocols to be used in regenerative medicine [457,458,459]. In recent times, the use of G-CSF-mobilized peripheral blood HSPCs has largely replaced bone marrow as a source of stem cells, although research has to go on to identify new agents or combinations, which may lead to more efficient stem cell mobilization aimed at entering clinical practice [460,461,462,463,464]. In particular, individualized techniques are required to enhance HSPC yields and to harvest adequate quantities of CD34^+^ cells, especially for patients whose cells mobilize poorly [59,465,466].

#### 9.1.2. Cytokine-Induced Ex Vivo Amplification and Differentiation of Erythroid Cells

**Figure 3:**
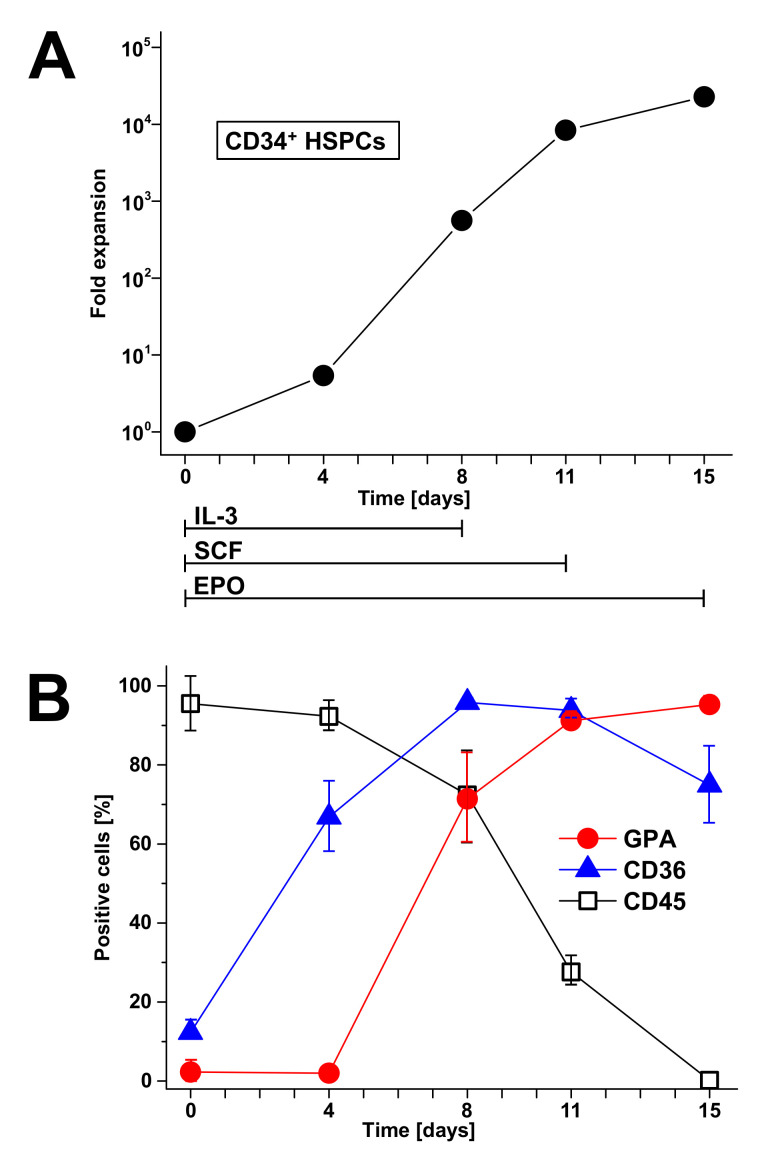
Cytokine-driven amplification of ex vivo cultured CD34^+^ HSPCs monitored in a time interval of 15 days (**A**) and the portrayal of cell surface markers of the developing erythrocytes (**B**). Cells were cultivated in the presence of interleukin (IL)-3, stem cell factor (SCF), and erythropoietin (EPO), which were applied from the initiation of cultivation at day 0 until day 8 (IL-3), day 11 (SCF), and the end of cultivation at day 15 (EPO). The graph shows the average cell expansion of HSPCs of three donors ([145], modified).

A number of protocols have been established for the isolation of CD34^+^ HSPCs from peripheral blood by immunomagnetic separation using iron beads coated with an anti-CD34 monoclonal antibody and the ensuing ex vivo propagation of erythroid cells (see Section 4). This strategy allows the differentiation and amplification of CD34^+^ HSPCs covering the various stages from early erythroblasts to reticulocytes (see Figure 1 and Section 3). Figure 3 portrays the ex vivo propagation and differentiation of erythropoietic cells over a period of 15 days obtained from three healthy donors based on recently obtained experimental data [145]. The ex vivo cell expansion starts with the inoculation of a cell culture with CD34^+^ HSPCs in conditioned medium supplemented with IL-3, SCF, and EPO as indicated in Figure 3A. IL-3, SCF, and EPO are added from the time point of initiation of culturing until days 8, 11, and 15, respectively. The cytokine-stimulated cultivation gave an average approximately 22.700-fold cell multiplication in the time period from day 0 to day 15. In order to monitor the course of differentiation in the ex vivo erythropoiesis model, flow cytometry analysis allows for the distinction of various types of early- and late-maturing blood cells by their surface-exposed marker proteins such as GPA, CD36, and CD45, as shown in Figure 3B. GPA is characteristic for erythroid cells, CD36 (also known as glycoprotein IV) is a specific marker of erythroid progenitor cells, which declines during terminal maturation to erythrocytes, and CD45 is expressed on HSPCs, erythroid cells at early stage of maturation, and myeloid cells. Starting with mobilized CD34^+^ HSPCs and monitoring the time course of altered cell surface marker proteins covers the various stages of erythroid differentiation and corroborates the ex vivo erythropoietic cell culture model (see Section 4.3).

#### 9.1.3. Morphological Alterations of Developing Erythrocytes

**Figure 4:**
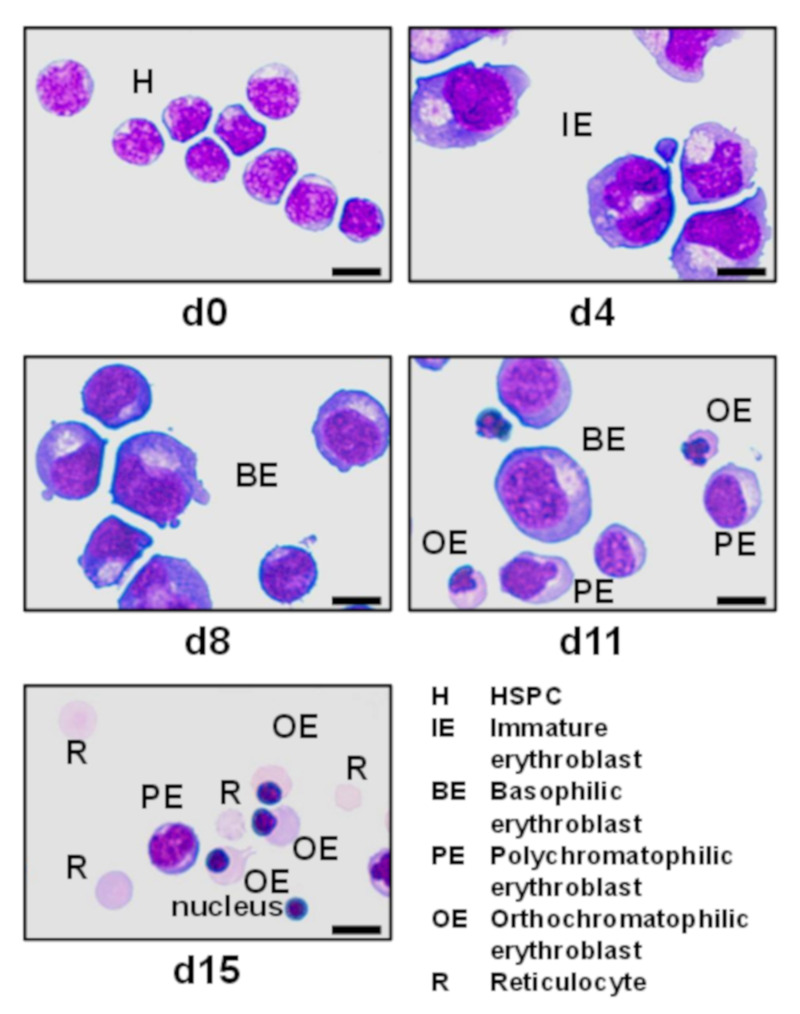
Cytospin preparations of adequate samples of HSPCs ex vivo propagated for a period of 15 days (d). Cells were obtained from donor 2 and stained with May–Grünwald–Giemsa solution; the bars correspond to 10 µm ([145], modified).

Adequate samples of cytospin preparations were performd with cell aliquots from ex vivo HSPCs cultured for 15 days [145] and are shown by way of an example of donor 2 in Figure 4. The May–Grünwald–Giemsa stain illustrates the morphological changes during erythropoietic differentiation. From day 0 to day 4, the uniformly stained HSPCs enter the first stage of differentiation of immature erythroblasts (proerythryoblasts) (see Figure 1 and Section 3). An increasing number of further matured basophilic erythroblasts is observed at day 8, followed by switching to the prevalence of polychromatophilic erythroblasts, which is accompanied by slowly rising orthochromatophilic erythroblasts and in a number of considerably reduced basophilic erythroblasts. The final stage of ex vivo erythropoiesis is attained at day 15 with the main transition into orthochromatophilic erythroblasts and enucleated reticulocytes.

#### 9.1.4. Synopsis of Morphological Shifts During Ex Vivo Erythropoiesis

**Figure 5:**
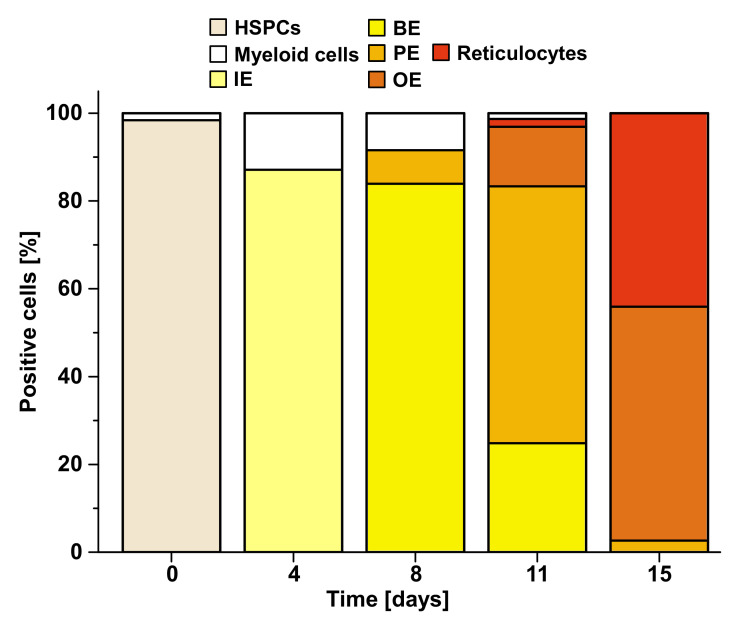
Quantitative assessment of ex vivo erythropoiesis of HSPCs during 15 days of development. Average cell counts from the different developmental stages (see Figure 4) were performed based on the May–Grünwald–Giemsa-stained erythrocyte progenitor cells of three donors ([145], modified).

In order to assess the magnitude of the ex vivo erythropoietic differentiation of HSPCs during 15 days of cultivation [145], the average cell counts of maturing erythroid cells obtained from three donors is shown as bar chart in Figure 5. Cell stainings of day 4 revealed a shift of HSPCs to 87.3% of immature erythroblasts, followed by a switch to mostly basophilic erythroblasts determined at day 8 amounting to 83.7% and indicating almost full commitment of HPSCs to the erythroid lineage. An increased percentage of 58.8% of hemoglobin-producing polychromatophilic erythroblasts was a characteristic feature of the cell samples on day 11, accompanied by newly emerging 13.7% of orthochromatophilic erythroblasts. The final stage of ex vivo erythropoietic differentiation at day 15 ended up with orthochromatophilic erythroblasts and reticulocytes, representing 53.6% and 43.8% of the cell culture, respectively. These data corroborate the suitability of ex vivo expansion to track the in vivo differentiation and maturation of erythropoietic cells.

### 9.2. Shiga Toxin-Mediated Damage of Erythroblasts During Erythropoiesis

**Figure 6:**
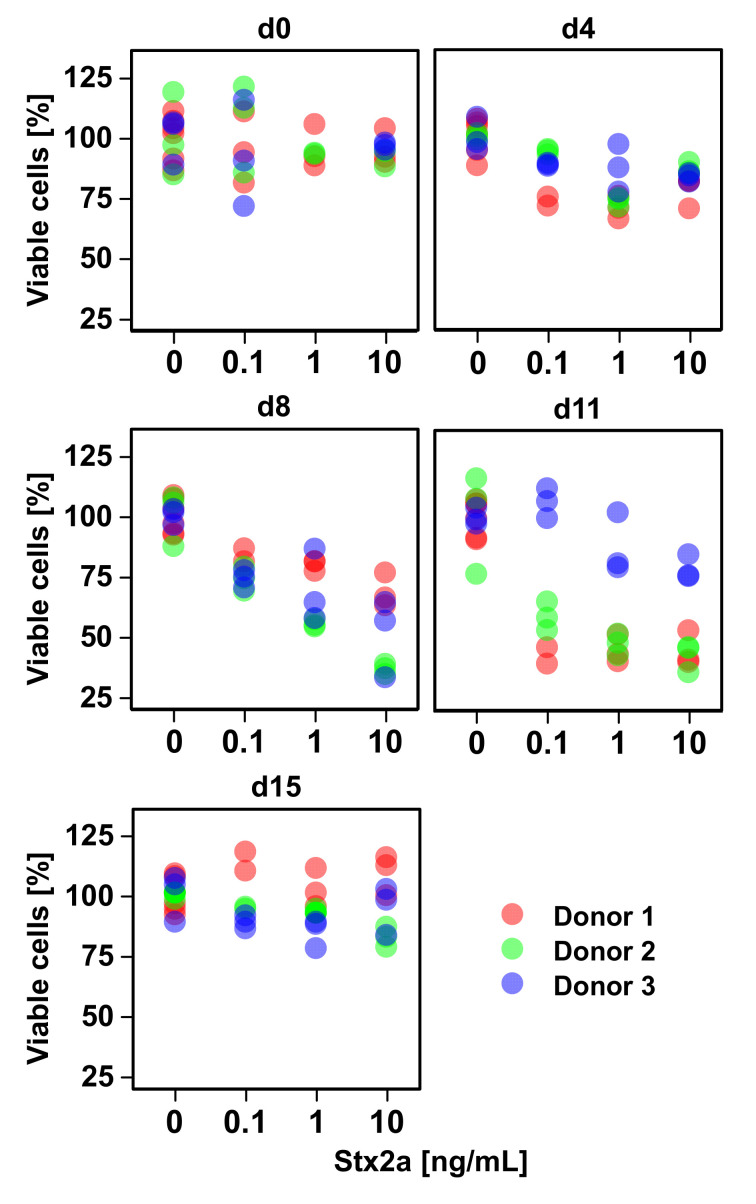
Loss in cell viability of ex vivo propagated erythrocyte progenitor cells caused by Stx2a at intermediate stages of maturation. Cell culture aliquots were taken from the different developmental stages during the cultivation of 15 days (d) (see Figure 4 and Figure 5) and exposed for 48 h to increasing Stx2a concentrations of 0.1 ng/mL, 1 ng/mL, and 10 ng/mL or kept in cell culture medium without toxin (control). Cell viability triple determinations were performed in relation to control cells and are provided as percent values. Colored full circles indicate the three donors: red marks donor 1, green donor 2, and blue donor 3 as indicated ([145], modified).

The knowledge of cytotoxic effects of EHEC-released Stxs toward HSPCs and their erythroid descandants is poor. Therefore, we asked for the possible direct cytotoxic action of clinically highly relevant Stx2a (see Section 7) to developing erythrocytes in the ex vivo erythropoietic cell culture model [145]. The extent of the Stx2a-caused impact on the cell viability upon challenging with toxin concentrations of 0.1, 1, and 10 ng/mL in the period of 15 days of ex vivo differentiation is displayed in Figure 6. A clear dose-dependent cell-damaging effect along with ascending concentrations of Stx2a was detected at day 8 and day 11 for the three individual donors. The Stx-responsible cell samples of day 8 comprise mainly basophilic erythroblasts, and those of day 11 harbor mostly polychromatophilic erythroblasts (see Figure 4 and Figure 5). On the other hand, the Stx-resistant cell samples of day 0 contain exclusively HSPCs and those of day 15 are solely composed of orthochromatophilic erythroblasts and reticulocytes (see Figure 4 and Figure 5). This demonstrates the preferential Stx2a-mediated injury of nucleated erythrocyte progenitor cells at intermediate stages of erythropoiesis, especially in the time interval corresponding to the basophilic and polychromatophilic differentiation. In striking contrast to this, undifferentiated HSPCs at the very early stage of development as well as orthochromatophilc erythroblasts and enucleated reticulocytes at the late stage of erythropoietic differentiation are highly refractory and almost resistant toward Stx2a.

### 9.3. Globo-Series Glycosphingolipids of Human Erythropoietic Cells

A brief retrospective view roughly compiles the present knowledge on the GSL expression during human stem cell differentiation and summarizes existing preliminary data regarding the GSL composition of developing human erythroid cells analyzed so far. Recently obtained new insights into the developmental changes of Gb3Cer and Gb4Cer content observed for ex vivo cultivated primary human erythropoietic cells throughout maturation finalizes this part of the review.

#### 9.3.1. Glycosphingolipid Expression during Human Stem Cell Differentiation

Owing to the very limited amounts of cell material available, studies directed at GSL analysis of human stem cells has in the past been mainly based on immunological assays and, hence, added up to the indirect detection of carbohydrate antigens on the cell surface using poly- or monoclonal antibodies [467]. In recent years, the knowledge regarding the precise structural characterization of GSLs and the determination of GSL dynamics in human embryonic and pluripotent stem cells has been vastly extended by biochemical and biophysical concepts based on latest progress in the design of improved analytical methodologies [166,468,469]. GSLs are principally considered marker molecules of various human stem cell types, including HSPCs [470]. Switching of the GSL core structure between the various GSL series indicated some exceptionally altered stage-specific GSL expression during human embryonic stem cell differentiation using flow cytometry and mass spectrometry analyses [471]. With the aim to bring human stem cells into clinical application, particularly for use in regenerative medicine and transplantation, the structural complexity of the GSL composition of stem cells has to be defined. The precise structural GSL analysis of human stem cells using antibody and lectin binding assays combined with mass spectrometry and proton NMR revealed a huge glycan diversity to be more complex than previously expected [472,473,474].

#### 9.3.2. Glycosphingolipid Expression of Human Erythroid Cells

The globo-series GSLs of primary erythropoietic cells are of particular interest from the medical microbiological viewpoint, because Gb3Cer (and to less extent Gb4Cer) are well-known functional and highly specific receptors of Stxs released by an emerging number of human-pathogenic EHEC strains (see Section 7). However, beyond the indirect detection of surface-exposed glycan structures of developing erythrocytes, detailed analyses of the GSL content of primary human erythropoietic cells are very limited in the literature. Early studies with a Gb4Cer-specific monoclonal antibody demonstrated the presence of globoside (Gb4Cer) on RBCs and erythroblasts, but not on erythroblast precursors (CFU-E, BFU-E), immature erythroblasts (proerythroblasts, see Figure 1) or on the cells of the proerythroblastic cell lines K562 and HEL [475] (see Section 6). Importantly, K562 cells expressed Gb4Cer upon the induction of maturation into erythroblasts [475]. This finding was inline with precedent GSL analyses of the Marcus group in the 1980s, who evidenced Lc3Cer and nLc4Cer as the characteristic neutral GSLs of K562 cells [189], while the team of Hakomori detected only trace quantities of Gb4Cer (and Gb3Cer) in ensuing studies of the K562 cell line [190] (for structures, see Figure 2).

#### 9.3.3. Glycosphingolipid Expression of Ex Vivo Amplified Primary Human Erythropoietic Cells

**Figure 7:**
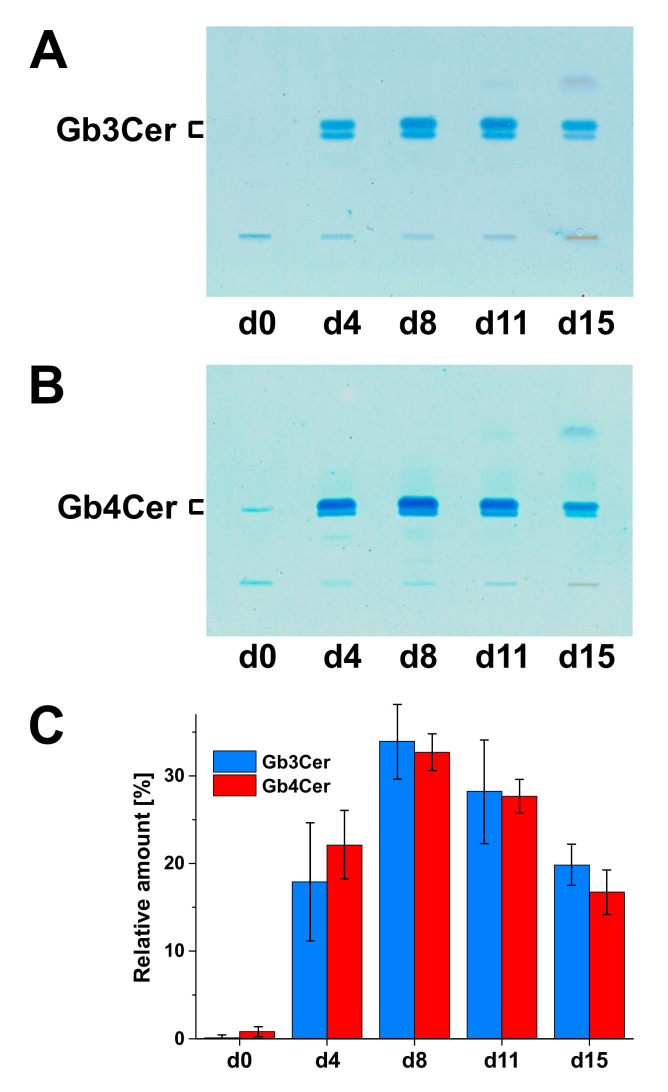
Immunochemical detection of Gb3Cer (**A**) and Gb4Cer (**B**) and portrayal of the dynamic change of Gb3Cer and Gb4Cer content during the ex vivo propagation of erythroid progenitor cells (**C**). The detection of TLC-separated Gb3Cer and Gb4Cer in lipid extracts of donor 2, which correspond to 1 × 10^6^ cells, each, was conducted with an anti-Gb3Cer and anti-Gb4Cer antibody, respectively. The bar chart depicts the averaged TLC scanning values of three donors showing the relative ascent and descent of Gb3Cer and Gb4Cer content during ex vivo erythropoiesis (see Figure 4 and Figure 5) ([145], modified).

The occurrence of Gb3Cer and Gb4Cer in erythrocyte-committed precursor cells and developing erythrocytes at various erythroblastic stages (see Figure 1) is of particular interest for the medical microbiologist, because globo-series GSLs are functional and specific receptors of Stxs (see Section 7). The analysis of developmental changes of GSLs of cells throughout erythropoiesis using a pure population of isolated precursor cells is feasible only to a limited extent. In continuation of our recently obtained data on the course of ex vivo erythropoiesis showing Stx2a-mediated cell damage of erythroblasts at intermediate developmental stages (see Figure 4, Figure 5 and Figure 6), we questioned the expression of Gb3Cer and Gb4Cer during erythropoietic differentiation. As a result, we were able to show for the first time the presence of Gb3Cer and Gb4Cer in erythropoietic cells during ex vivo maturation, as depicted in Figure 7, using lipid extracts obtained from cell samples of donor 2 as a representative example [145]. The thin-layer chromatography (TLC) overlay analysis revealed the absence of Gb3Cer in HSPCs at day 0 (Figure 7A), a clear detection of a Gb3Cer-positive double band at day 4, further increase of Gb3Cer double-band intensity at day 8, followed by a slight gradual decrease on day 11 and stronger decline on day 15. A very similar rise and fall of Gb4Cer-positive double bands throughout erythrocytic development was detected with the anti-Gb4Cer antibody (Figure 7B). The averaged densitometric TLC quantification of the scan values of three donors is summarized in the bar graph shown in Figure 7C indicating a maximal content of Gb3Cer and Gb4Cer in the time frame of ex vivo erythropoiesis on day 8 and day 11, when the dominance of basophilic and polychromatophilic erythroblasts, respectively, was apparent in the cell samples.

### 9.4. Shiga Toxin 2a-Mediated Cell Damage and Toxin Receptors of Ex Vivo-Propagated Erythropoietic Cells

**Figure 8:**
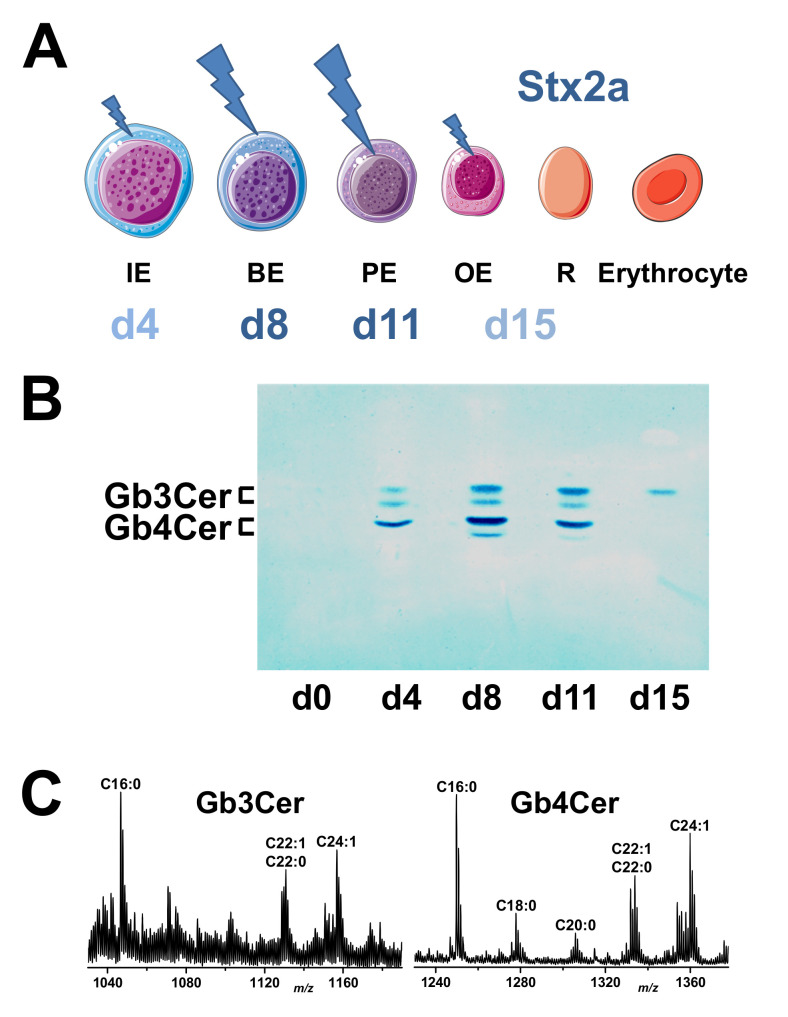
Scale of Stx2a-mediated cell damage over time during 15 days of ex vivo expansion of erythroid cells (**A**) and Stx2a TLC attachment toward Gb3Cer and Gb4Cer of the developing erythrocytes (**B**) together with mass spectra showing the various lipoforms of Stx2a-binding Gb3Cer and Gb4Cer (**C**). (**A**) The figure was adapted from SERVIER MEDICAL ART (https://smart.servier.com). IE, immature erythroblast (proerythroblast); BE, basophilic erythroblast; PE, polychromatophilic erythroblast; OE, orthochromatophilic erythroblast; R, reticulocyte (see Figure 1). This panel summarizes the data of the May–Grünwald–Giemsa-stained cell samples shown in Figure 4 and Figure 5, and the viability assays of cellular Stx2a exposure depicted in Figure 6. (**B**) The Stx2a overlay assay was performed with aliquots of lipid extracts prepared from cell samples of donor 2, each equal to 1 x 10^6^ cells, and served as a representative example of three donors. (**C**) The Gb3Cer and Gb4Cer mass spectra were produced from extracts of TLC overlay-detected Gb3Cer and Gb4Cer (see Figure 7) of cell samples from donor 2 corresponding to day 8 of the ex vivo cell culture (for structures, see Figure 2) ([145], modified). The respective ceramide moieties harbor sphingosine (d18:1) as the invariable portion linked with a fatty acid variable in carbohydrate chain length from C16 up to C24, as indicated in the spectra.

A résumé of the time frame of the Stx2a-caused injury of erythroid progenitor cells during 15 days of ex vivo propagation together with binding patterns of Stx2a-recognized Gb3Cer and Gb4Cer species and their structural features explored by mass spectrometric analysis is given in Figure 8. The pictorial representation in Figure 8A summarizes the progress over time and magnitude of Stx2a-mediated cellular damage emphasizing higher-ranking damage to nucleated basophilic and polychromatophilic erythroblasts when compared to immature (proerythroblasts) and orthochromatophilic erythroblasts [145].

The TLC overlay detection using Stx2a combined with an anti-Stx2 antibody demonstrates pronounced binding of the toxin toward Gb3Cer and Gb4Cer at day 8 and day 11 of the ex vivo cell culture [145]. Remarkably, the Gb4Cer upper band of the doublet exhibited strongest positive binding intensity compared to less intensively stained Gb3Cer double bands. Although less sensitive than the antibody-mediated detection, as can be deduced from the color intensities of the antibody-positive bands (see Figure 7), receptor detection by Stx2a (Figure 8B) is in agreement with the immunochemical detection, showing the enhanced content of Gb3Cer and Gb4Cer at day 8 and day 11, lowered content at day 4 and day 15, and absence of Stx-binding GSLs in the beginning of the cell culture at day 0. Bearing in mind that Stx2a exhibits an inferior binding strength toward Gb4Cer in comparison to Gb3Cer [316], its stronger staining intensity toward Gb4Cer observed in the toxin TLC overlay assay suggests a significantly higher concentration of Gb4Cer versus Gb3Cer in the developing erythrocytes. Due to limitation in GSL material, an orcinol stain of TLC-separated GSLs allowing a direct comparative analysis (in contrast to indirect immunochemical and Stx-mediated detection) was not feasible [145]. However, with the exception of day 0, highly sensitive mass spectrometry analysis was successful in terms of the structural characterization of the various Gb3Cer and Gb4Cer lipoforms [145]. An example is provided in Figure 8C showing mass spectra of Gb3Cer and Gb4Cer, which were obtained from extracts of the immunostained Gb3Cer and Gb4Cer double bands of samples derived from donor 2 (see Figure 7A,B, respectively) at day 8 of the ex vivo cell culture. Gb3Cer and Gb4Cer lipoforms harboring sphingosine (d18:1) as the constant moiety of the ceramide lipid anchor and variable fatty acids ranging from C16 to C24 chain length were detected during the superior Stx2a-responsive phase of the 15 days lasting ex vivo cultivation. Failure of mass spectrometric detection in GSL preparations of day 0 was in line with the negative results of antibody- and Stx-mediated TLC overlay detection (see Figure 7).

## 10. Conclusions and Outlook

The data presented in this review demonstrate that Stx2a, a clinically highly relevant Stx subtype, has a detrimental effect on developing erythrocytes propagated in a human ex vivo erythropoiesis cell culture model. Using low doses of Stx2a, the toxin-induced loss in cell viability confines to intermediate developmental stages and is most likely restricted to nucleated erythroid cells. We hypothesize that hemolytic anemia during HUS, by definition the loss of erythrocytes due to their mechanical deformation and disruption in the bloodstream, arises not only from intravascular cell damage due to passing through occluded microvessels, but also from the extravascular impairment of erythrocyte progenitors in the bone marrow via direct damage by Stx2a leading to “non-hemolytic” anemia. Moreover, a possible “supportive” effect of EHEC-Hly regarding the manifestation of anemia in EHEC-associated HUS should be given more attention in coming studies dealing with erythropoietic cells. EHEC-Hly might presumably target especially developing erythrocytes in the late stages of differentiation, which are largely refractory to the cytotoxic action of Stx. These stages are the orthochromatophilic erythroblasts and enucleated reticulocytes (see Figure 8) and investigation of their response toward EHEC-Hly is a challenging approach for pending future experiments. Thus, the question remains whether EHEC-Hly may act as a “helpful dude” for Stx perhaps having a cumulative effect on the serious injury of the human erythropoietic system that should be analyzed in a timely manner. Unquestionably, the results of our ex vivo cell cultures provide reasonable evidence for a possible correlation with Stx-induced hemolysis and subsequent hemolytic anemia during EHEC infections in vivo. Our hypothesis is further underlined by previous and current complementary literature from interdisciplinary and closely related research fields videlicet glycobiology, stem cell biology, cell culture technology, medical microbiology, and biophysics. Thus, from our viewpoint, it seems rather unlikely that mechanical stress in narrowed vessels alone could be the reason for the anemic condition in EHEC-infected patients. Rather, anemia occurring during HUS seems to be a multifaceted event whereby multiple processes might be involved in the dramatic loss of circulating erythrocytes. Thus, in our opinion, HUS-associated anemia can be caused either by an increased red blood cell breakdown, but it might be also caused by a decreased red blood cell production in the bone marrow, resulting in anemic conditions in EHEC patients. In this context, it deserves to be mentioned that isotope-labeled verotoxin 1 and 2 (synonymous to Stx1a and Stx2a, respectively) applied to mice were localized in the murine bone marrow of the spine, long bones, and ribs, indicating the presence of Stx receptors, although the type of Stx-binding cells has not been determined [476]. Collectively, the sum of data vividly underscores our view as emphasized in the provocative title on the “Valid Presumption of Shiga Toxin-Mediated Damage of Developing Erythrocytes in EHEC-Associated Hemolytic Uremic Syndrome”.

Future research on Stx-mediated HUS is in want of a mouse model that conforms to the human scenario of EHEC infections and recapitulates the pathogenesis caused by EHEC as a whole. Various mouse models that simulate one or more events of EHEC infection and disease have been developed [477]. They can be used to monitor EHEC colonization, disease, pathology, or combinations of these features. However, murine models of EHEC toxemia do not develop thrombocytopenia and, therefore, do not present with Stx-induced HUS [399]. Thus, unfortunately there is not yet any one mouse model that fully mimics the spectrum of EHEC illness [222]. A different GSL status, i.e., the absence or low content of the canonical Stx receptors Gb3Cer and/or Gb4Cer in mouse endothelial cells versus the human counterparts, might be just one among many other reasons for this discrepancy. On the other hand, piglets are known to exhibit a similar GSL repertoire similar to humans in terms of globo-series GSLs occurring in many organs, including the kidney and the brain [478]. This makes piglets suitable to further study the pathophysiology of EHEC-induced HUS. Gnotobiotic piglets orally infected with EHEC wild-type isolates developed intestinal and extraintestinal manifestations of EHEC diseases, including thrombotic microangiopathy in the kidneys, the morphologic hallmark of HUS in humans [479]. However, the reservation must be made that the breeding and maintenance of large animal models, such as the gnotobiotic piglet, require considerable veterinary skill, space, and financial support [477]. Although mouse models do not fully mimic human infection, strategies for the evaluation of novel EHEC therapeutics are evolved in murine models of infection [480]. Promising vaccine candidates offering high prospects are EHEC-specific polysaccharide conjugates that induced high levels of anti-LPS antibodies in humans with bactericidal activities against *E. coli* O157 or constructs using the B subunit of Stx that elicited both bacterial and toxin-neutralizing antibodies in mice [481]. Monoclonal antibodies raised against O-polysaccharide–protein conjugates with an exceptional degree of specificity and relative affinity against *E. coli* O157 and O145 serogroups have been recently developed [482]. B subunit-specific murine monoclonal antibodies that strongly neutralize the toxicity of Stx2 have been generated as well, which may serve as the basis for generating mouse–human chimeric Stx2-neutralizing antibodies [483]. Such protective efficacy of chimeric anti-Stx1 and anti-Stx2 monoclonal antibodies has been demonstrated shortly afterwards, which could be used therapeutically for the prevention or treatment early in the development of HUS [484]. Recombinant antibody fragments that recognize and neutralize Stx or camelid single chain antibodies against Stx with therapeutic potential against HUS are other candidates for future clinical applications, respectively [485,486,487]. Furthermore, plant-based recombinant secretory IgA represents an innovative approach applicable as an agent for oral passive immunotherapy [488,489]. Based on the interaction of Stx2 with the complement system [251], the C5-complement inhibiting monoclonal antibody Eculizumab was therapeutically employed as rescue therapy for EHEC–HUS patients, which had seizures or were in a stupor or coma [490]. In a number of studies, Eculizumab showed positive clinical improvement after treatment in severe EHEC-HUS with progressive neurological improvement that often lead to serious long-term disabilities [491]. In particular, the early use of Eculizumab appeared to improve neurological outcome suggesting that prophylactic antibody therapy before the development of neurological symptoms could be advantageous [490]. However, future proper randomized controlled trials are urgently needed to resolve the debate as to whether Eculizumab can be a prophylactic treatment for the prevention of extraintestinal complications or curing of EHEC–HUS [492]. Among potential glycoconjugate-based neutralizers of Stx that could capture free Stx within the gut or the circulation and impede or at least attenuate the toxin’s action either locally or systemically, respectively [493,494,495], semisynthetic neoglycolipids with glycans derived from fruit or vegetable pectins might be suitable as a supportive measure in EHEC diseases. Embedded in lipid vesicles, termed “glycovesicles”, pectin-derived and Gb3-carrying neoglycolipids have shown their capacity as multivalent inhibitors of the cytotoxic action of various Stx subtypes in vitro [496,497]. As a dietary supplement, neoglycolipids can be orally applied as glycovesicles or encapsulated, e.g., in pectin–alginate particles, to provide gastroresistance [498] and could improve EHEC and HUS patient outcomes. Unfortunately, despite decades of work unraveling the mechanisms of Stx toxicity, there are currently no available causative therapeutics to prevent or treat EHEC-associated HUS, while therapy is limited to supportive care based mainly on alleviating symptoms [254,305,499]. Last but not least, the emergence of new strains with rapidly aggressive virulence makes clinical and research initiatives in this field a high public health priority [278,279,399]. The stepping up of efforts directed toward the development of Stx therapeutics beyond neutralization is an additional challenge [500] with large global incidence for the years ahead to combat or, preferably, to prevent EHEC epidemic outbreaks [305].
